# Inflammation-dependent downregulation of miR-532-3p mediates apoptotic signaling in human sarcopenia through targeting *BAK1*

**DOI:** 10.7150/ijbs.41641

**Published:** 2020-02-24

**Authors:** Fa-Xiu Chen, Yi Shen, Yang Liu, Hai-Feng Wang, Chen-Yu Liang, Ming Luo

**Affiliations:** 1 Department of Geriatrics, Tongji Hospital, Tongji University School of Medicine, Shanghai 200065, China.; 2 Department of Geriatrics, Jiangxi Provincial People's Hospital Affiliated to Nanchang University, Nanchang 330006, Jiangxi, China.

**Keywords:** sarcopenia, microRNA, miR-532-3p, BAK1, NFKB1, TLR4

## Abstract

Inflammation and apoptosis are considered as two major pathological causes of human sarcopenia. The current understanding based on different models recognizes that apoptosis does not trigger inflammation, while emerging evidence indicates that inflammation can induce apoptosis. Here, we provide solid evidence to suggest that the inflammation-dependent downregulation of miR-532 causes apoptosis through targeting a proapoptotic gene *BAK1* (BCL2 antagonist/killer 1). To identify miRNAs and genes that are aberrantly expressed in the muscle tissues of sarcopenia patients, we conducted two independent microarray analyses. In total, we identified 53 miRNAs and 69 genes with differential expression levels. Of these aberrantly expressed miRNAs, miR-532-3p showed the most obvious changes in sarcopenia tissues, and more importantly, it can be repressed by the well-known inflammatory inducer lipopolysaccharide (*LPS*) *in vitro*. According to gene-based microarray results and the predicted targets of miR-532-3p, we presumed that *BAK1* was a putative target of miR-532-3p. Further *in vitro* and *in vivo* analyses verified that miR-532-3p could directly bind to the three prime untranslated region (3'-UTR) of *BAK1* through the seed sequence CUCCCAC. In addition, we found that NFKB1 (also known as p50), a subunit of the transcription factor NF-κB (*nuclear factor* kappa-light-chain-enhancer of activated B cells), could specifically bind to the promoter region of miR-532-3p and repress its expression. Further analysis revealed that the activation of TLR4 (Toll-like receptor 4) signaling led to the translocation of p50 from the cytoplasm to the nucleus, where it repressed miR-532-3p expression and thus led to an increase of *BAK1*. The accumulated BAK1 activated its downstream apoptotic signaling pathways and resulted in apoptosis, eventually causing the pathogenesis underlying sarcopenia. Overall, our results uncovered a new mechanism by which the inflammation-dependent downregulation of miR-532-3p contributed to the pathogenesis of sarcopenia through mediating *BAK1* expression.

## Introduction

During the aging process, humans generally experience the loss of skeletal muscle, and the pathological process of extreme muscle loss is known as sarcopenia 1, 2. Growing evidence suggests that the disruption of anabolic (e.g., diminishing levels of growth hormone and testosterone) and catabolic signals (induction of proinflammatory cytokines) can contribute to the pathogenesis of sarcopenia 1-3. Generally, an increase in proinflammatory cytokines promotes muscle wasting stimulates protein catabolism and suppresses muscle synthesis 4, 5. Elevated levels of proinflammatory cytokines have a negative correlation with muscle strength and muscle mass 6, 7. The clinical data from a large population indicate that elderly sarcopenia patients have significantly elevated concentrations of proinflammatory cytokines such as IL-6 (*Interleukin 6*) and TNF-α (*Tumour Necrosis Factor-Alpha*) 8-10. Moreover, a 10-year clinical tracking survey also suggested that the serum concentrations of IL-1β, IL-6 and TNF-α are closely associated with morbidity and mortality in older sarcopenia patients 11. The inflammatory response is a protective mechanism by which immune cells respond to external stimuli or intracellular damage 12. Signaling pathways involved in inflammation have been well characterized. One of the most important involved pathways is the TLR4/NF-κB (Toll-like Receptor 4/*Nuclear Factor* Kappa-light-chain-enhancer of Activated B cells) signaling axis 13. Initially, the CD14-TLR4-MD2 complex on the cell surface is stimulated by lipopolysaccharide (*LPS*) 13. Then, the activated TLR4 associates with TIRAP (Toll/Interleukin-1 Receptor Domain-containing Adapter Protein), TRAM (Translocation Associated Membrane Protein), TICAM1 (Toll-like Receptor Adaptor Molecule 1), MyD88 (Myeloid Differentiation Primary Response 88), and BTK (Bruton Tyrosine Kinase) to form a complex that engages the E3 ligase TRAF6 (TNF Receptor-associated Factor 6) to modify the IKK (I-kappa-B kinase) complex 13-15. The IKK complex phosphorylates the transcription factor NF-κB to cause it to translocate from the cytoplasm to the nucleus, where it can activate the expression of multiple proinflammatory cytokines, such as IL-1β, IL-6 and TNF-α 13-15. Although elevated levels of proinflammatory cytokines have been identified in sarcopenia, the upstream signaling pathways that control proinflammatory cytokine production are still obscure.

Apoptosis is a kind of programmed cell death that is often associated with degenerative diseases, including sarcopenia 16, 17. Growing evidence has demonstrated the activation of apoptotic signaling in the pathological process underlying sarcopenia 17. Using rat and mouse models, different research groups have found increased levels of BAX (BCL2-associated X Protein) and the activation of caspase-dependent signaling in degenerative muscle tissues 17, 18. Currently, two independent apoptosis signaling pathways, the intrinsic pathway and the extrinsic pathway, have been well characterized 19, 20. The intrinsic pathway, also known as the mitochondrial pathway, is activated when cells release cytochrome* c* from mitochondria through the actions of BAX and BAK1 (Bcl-2 homologous Antagonist/Killer 1) 19, 20. The released cytochrome* c* binds to APAF1 (Apoptotic Protease Activating Factor 1), ATP and pro-caspase-9 to form an apoptosome complex, which mediates downstream caspase cascades and eventually leads to apoptosis 19, 20. The extrinsic pathway senses extracellular signals through cell-surface TNF (tumour necrosis factor) receptors, which in turn bind to TNF receptor-associated death domain (TRADD) and Fas-associated death domain (FADD) to activate downstream caspase cascades 19, 20. Although molecules that are involved in apoptosis have been shown to be activated during the pathological process underlying sarcopenia, it is not well known how signaling is activated.

MicroRNAs (miRNAs) are a subclass of naturally occurring non-coding RNAs with an average length of 18-25 nucleotides 21, 22. Generally, miRNAs bind to the three prime untranslated regions (3'-UTR) of their targets to induce mRNA degradation and translational repression 21, 22. In recent years, miRNAs have been shown to play important roles in the pathogenesis of different diseases, including sarcopenia 23-25. Using miRNA microarray or next-generation sequencing methods, researchers have identified a number of differentially expressed miRNAs involved in muscle aging processes 26, 27. For instance, Hamrick and colleagues identified 36 downregulated (e.g., miR-181a, miR-434, miR-382, miR-455, miR-124a, and miR-221) and 21 upregulated miRNAs (e.g., miR-206, miR-7, miR-542, miR-468, and miR-698) in quadriceps muscle tissues from old mice compared to the levels in young mice 26. Kim and colleagues found 34 differentially expressed miRNAs (e.g., miR-34a-5p, miR-146a-5p, miR-92b-3p, miR-155-5p, miR-203-3p, miR-337-3p, miR-434-3p, miR-434-5p, miR-136-5p, and miR-148a-3p) in gastrocnemius muscle from old mice compared to the levels in young mice 27. Some of these miRNAs have been identified to target important molecules and pathways involved in the aging process 28. For instance, miR-195 can target *SIRT1* (Sirtuin-1) and *TERT* (Telomerase Reverse Transcriptase) 29. Four miRNAs, including let-7, miR-29, miR-125b and miR-143-3p, can target *IGF1* (Insulin-like Growth Factor-1) and *CDK6* (Cyclin Dependent Kinase 6) to inhibit myogenesis 30, 31. Several miRNAs, including miR-26a, miR-146b, miR-431 and miR-675, can inhibit the transforming growth factor β (TGF-β) signaling pathway to enhance muscle regeneration 30, 32, 33. Thus, miRNAs have emerged as important biomarkers of sarcopenia and intervention targets for the therapy of muscle aging.

Although multiple miRNAs have been found to play critical roles in the pathogenesis of sarcopenia, none of them target molecules that are involved in inflammation and apoptosis. Given that inflammation and apoptosis are two major causes that contribute to the pathogenesis of sarcopenia, we aimed to identify miRNAs involved in these two processes. For this purpose, we conducted a miRNA array assay using muscle tissues from older sarcopenia patients and healthy controls. In total, we identified 53 aberrantly expressed miRNAs. Of these, miR-532-3p was the most significantly downregulated miRNA in the sarcopenia patient samples and was shown to be regulated by LPS using an *in vitro* assay. We focused our study on revealing the downstream targets of miR-532-3p and the mechanism underlying its downregulation. Our results support a model in which TLR4/NF-κB axis signaling negatively regulates the expression of miR-532-3p and causes its downregulation in sarcopenia. The downregulated miR-532-3p reduces its inhibition of *BAK1*, causing the activation of BAK1-mediated downstream events and eventually leading to apoptosis.

## Materials and Methods

### Cell culture, transfection and treatment

Human skeletal muscle myoblast cell lines HSMM-1, HSMM-1 and LHCN-M2 were obtained from Lonza (Basel, Swiss, #CC2580), Applied Biological Materials Inc. (Richmond, BC, Canada, #T0033), and Evercyte (Vienna, Austria, #LHCN-M2), respectively. Cells were grown in DMEM medium (Sigma-Aldrich, St. Louis, MO, USA, #D5546) supplemented with 10% fetal bovine serum (FBS) (GE Healthcare, Waukesha, WI, USA, #SH30070.03EH) and 1% penicillin-streptomycin *antibiotic solution* (Thermo Fisher Scientific, Waltham, MA, USA, #15070063) and incubated in 5% CO_2_ at 37°C. The transfection of siRNAs and plasmids was performed according to the Dharmafect1 transfection protocol (T-2001-03). The siRNAs used in this study included siNFKB1 (#S9504 and #S9506), si-c-Jun (#7659 and #S7660), si-c-FOS (#S5339 and #S5340), and siSTAT1 (#S277 and #S288), which were purchased from the Thermo Fisher Scientific company. The plasmids including pCDNA3-2×Flag-NFKB1, pCDNA3-2×Flag-c-Jun, pCDNA3-2×Flag-c-FOS, and pCDNA3-2×Flag-STAT1 were constructed into the pCDNA3-2×Flag empty vector between BamHI and EcoRI sites using their corresponding coding sequences. The miRNA-532-mimic and anti-miR-532 were purchased from Shanghai GenePharma company. After incubation at 37°C for 48 h, the cells were washed with PBS (Phosphate Buffered Saline) buffer and then collected for RNA and protein isolation. For LPS and IL-1β treatments, cells grown to approximately 80% confluence were respectively treated with different concentrations of LPS (0, 50, 100 and 200 ng/mL) and IL-1β (0, 1, 5 and 25 ng/mL) for 6 h, followed by cell collection and processing for the required experiments.

### Measurement of serum cytokines

Venous blood samples were collected from 24 healthy/young volunteers and 24 sarcopenia patients (ages 55-82) who underwent treatment in the Department of Geriatrics, Jiangxi Provincial People's Hospital from 2011-2016. After collection, the blood samples were subjected to centrifugation at 1000 × *g* for 8 min to harvest the serum. The resulting serum was used to measure the concentrations of different cytokines, including IL-1β (#ΒΜS224-2), IL-4 (#ΒΜS225-2), IL-6 (#EH2IL6), IL-13 (#ΒΜS213-3), IL-15 (#ΒΜS2106), and TNF-α (#ΒΜS223ΗS) using ELISA kits, which were all purchased from the Thermo Fisher Scientific company.

### Tissue collection

Twenty-four-paired healthy and degenerative muscle samples (from the lateral mass of the quadriceps) were collected from 24 young patients (average age 26.3±3.1, all male) and 24 older sarcopenia patients (average age 64.3±6.2, all male). All of these individuals had sustained severe bone injuries and required surgical treatment. All participants provided written informed consent that was reviewed and approved by the ethical board of Jiangxi Provincial People's Hospital.

### MicroRNA isolation, microarray and qRT-PCR analyses

Muscle tissues were used for miRNA isolation with a RNeasy Mini Kit (QIAGEN, Shanghai, China, #217004) according to the manufacturer's protocol. After determining the miRNA quality, 200 ng miRNA was used for microarray analysis with a Human MiRNA Microarray Kit (Agilent, Santa Clara, CA, #G4471A-029297) according to the manufacturer's protocol. The slides were scanned with an Agilent G4900DA SureScan Microarray Scanner System. The expression of 6 differentially expressed miRNAs, including miR-17-3p, miR-34a-3p, miR-107, miR-126-5p, miR-192-3p, and miR-532-3p, were examined by qRT-PCR using TaqMan miRNA assays. The assay IDs of these miRNAs were 477932, 478047, 478254, 480908, 478741, and 478336, respectively. The expression of these miRNAs in the same healthy control sample was defined as one-fold, and their expression in other samples was normalized to this control.

### Total RNA isolation, microarray and qRT-PCR analyses

Cells and muscle tissues were subjected to total RNA isolation using TRIzol reagent (Sigma-Aldrich, #T9224) according to the manufacturer's protocol. A total of 500 ng of each RNA sample was labelled using a Two-Colour Low Input Quick Amp Labelling Kit (Agilent, #5190-2306) according to the manufacturer's protocol. The resulting RNA was subjected to microarray analysis using a SurePrint G3 Human Gene Expression 8 × 60K v2 kit (Agilent, #G4851B) according to the manufacturer's protocol. After hybridization and washing, the slides were scanned with an Agilent G4900DA SureScan Microarray Scanner System. The expression of individual genes was determined by qRT-PCR using a SYBR Green PCR Master Mix Kit (Thermo Fisher Scientific, #4309155) following a previously described protocol 34. The expression of genes in the control sample was defined as one-fold, and their expression in other samples was normalized to the controls. The qRT-PCR procedures included 95°C 2 min, and 40 cycles of 95°C 30 sec and 68°C 30 sec. The primers used in this study are listed in Supplementary Table-1.

### Western blotting

The western blotting procedure was performed as described previously 34. Briefly, cells and muscle tissues were lysed with RIPA buffer (Thermo Fisher Scientific, #89900) supplemented with protease inhibitor cocktail (Sigma-Aldrich, #S8820). The cytoplasmic and nuclear fractions were isolated using the NE-PER^TM^ Nuclear and Cytoplasmic Extraction Kit (Thermo Fisher Scientific, #78833) according to the manufacturer's protocol. Equal amounts of protein were separated in 12% SDS-PAGE gels and transferred to polyvinylidene difluoride (PVDF) membranes (Thermo Fisher Scientific, #LC2005), followed by incubation with primary antibodies including anti-BAK1 (Thermo Fisher Scientific, #MA5-32111), anti-caspase-3 (Abcam, Cambridge, MA, USA, #ab4051), anti-caspase-9 (Abcam, #ab25758), anti-p50 (Abcam, #ab7549), anti-p65 (Abcam, #ab32536), anti-TLR4 (Sigma-Aldrich, #SAB3500765), anti-MyD88 (Abcam, #ab2064), anti-TRAF6 (Sigma-Aldrich, #HPA020599), anti-HDAC1 (Sigma-Aldrich, #AV38530), anti-STAT1 (Abcam, #ab3987), anti-c-FOS (Abcam, #ab190289), anti-c-JUN (Abcam, #ab105186), anti-β-Actin (Sigma-Aldrich, #A2228) and anti-GAPDH (Abcam, #ab9483). After probing with secondary antibodies, the protein signals were examined using enhanced chemiluminescence (ECL) reagents (Sigma-Aldrich, #GERPN2016). The protein signals were quantified using Image J software and the level of each protein in controls was defined as one-fold.

### Pharmacological treatments

Cells were treated with different chemicals, including 10 μM 5-aza-2′-deoxycytidine (AZA, a DNA methylation inhibitor), 5 μM trichostatin A (TSA, a histone acylation activator), 1 μg/mL CLI-095 (a TLR4 inhibitor), and 50 μM NBP2-29328 (a MyD88 inhibitor) for 6 h at 37 °C, respectively. Cells were then collected and used for the required experiments.

### Chromatin immunoprecipitation (ChIP) assays

ChIP assays were performed according to a standard protocol. Briefly, cells (5 × 10^7^) were washed once with cold PBS and then crosslinked with 1% formaldehyde for 10 min at 37°C. The resulting cells were used for ChIP assays with a High-Sensitivity Kit (Abcam, #ab185913). The antibodies used in this assay included anti-p50, anti-STAT1 and IgG (negative control). The primer sequences used for the qRT-PCR assay were: 5'-TGTGCTGCACCCGTTAACTT-3' (forward) and 5'-CATGTTCTCACTCATAGGTGG-3' (reverse).

### Statistical analysis

All experiments in this study were performed in triplicate. The data were analysed using two-sided Student's* t* tests. The significance levels were set at *P* < 0.05 (*), *P* < 0.01 (**) and *P* < 0.001 (***).

## Results

### Sarcopenia patients had elevated proinflammatory cytokine levels

**Figure 1:**
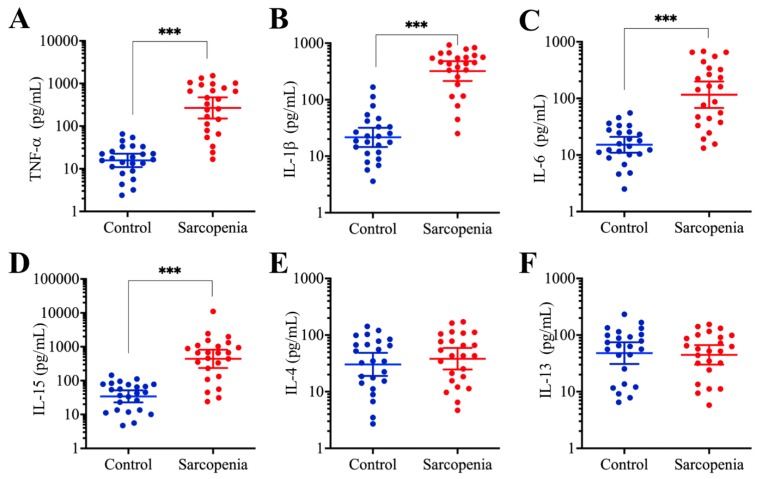
** The concentrations of proinflammatory cytokines were significantly increased in sarcopenia patients. Serum levels of cytokines, including TNF-α (A),** IL-1β **(B)**, IL-6 **(C)**, IL-15 **(D)**, IL-4 **(E)**, and IL-13** (F)**, were measured using ELISA assays in blood samples collected from 24 healthy and 24 sarcopenia patients. *** P < 0.001.

Inflammation is considered an important contributor to sarcopenia 11. To monitor changes in the serum concentrations of proinflammatory molecules in aged individuals, we conducted a five-year clinical survey in which we examined the serum levels of several important cytokines, including IL-1β, IL-4, IL-6, IL-13, IL-15, and TNF-α, to observe their association with the pathogenesis of sarcopenia. The average levels of the four proinflammatory cytokines, including IL-1β, IL-6, IL-15, and TNF-α, were significantly increased in sarcopenia patients compared to those in young/healthy volunteers, while the concentrations of two other cytokines, IL-4 and IL-13, were not changed (Figure 1). The specific serum concentrations in controls and sarcopenia patients, respectively, were as follows: for TNF-α, 16.4 pg/mL (range 2.84-88.59) compared with 277.13 pg/mL (range 23.65-1215.4, *P* < 0.001; Figure 1A); for IL- β, 22.62 pg/mL (range 3.82-183.54) compared with 342.67 pg/mL (range 26.78-905.46, *P* < 0.001; Figure 1B); for IL-6, 15.16 pg/mL (range 2.66-55.73) compared with 115.66 pg/mL (range 13.16-715.43, *P* < 0.001; Figure 1C); for IL-15, 32.55 pg/mL (range 4.25-155.65) compared with 319.26 pg/mL (range 21.85-1342.63, *P* < 0.001; Figure 1D); for IL-4, 31.66 pg/mL (range 2.95-157.84) compared with 38.52 pg/mL (range 3.84-191.91, *P* = 0.43; Figure 1E); for IL-13, 48.66 pg/mL (range 7.15-275.43) compared with 41.57 pg/mL (range 5.22-135.24, *P* = 0.67; Figure 1F). Given that the majority of proinflammatory cytokine genes are controlled by TLR4/NF-κB axis signaling, we speculated that this pathway would be activated in the sarcopenia patient samples. To verify this hypothesis, we determined the protein levels of critical components of the TLR4/NF-κB signaling axis, including TLR4, MyD88, TRAF6, p50 and p65. As shown in Supplementary Figures 1A and 1B, we found that the protein levels of TLR4, MyD88 and TRAF6 were significantly increased in three sarcopenia muscle samples, while those of the two NF-κB subunits, p50 and p65 were not increased. Given that many publications have shown that NF-κB subunits translocate from the cytoplasm to the nucleus when they are activated, we continued to examine their levels in the cytoplasmic and nuclear fractions. We found that the levels of both p50 and p65 were decreased in the cytoplasmic fractions, while they were increased in the nuclear fractions from all three sarcopenia muscle samples (Supplementary Figures 1C and 1D). These results further confirm that inflammation is a common phenomenon in sarcopenia patients.

### MicroRNA-532-3p was significantly downregulated in muscle tissues from sarcopenia patients

**Figure 2:**
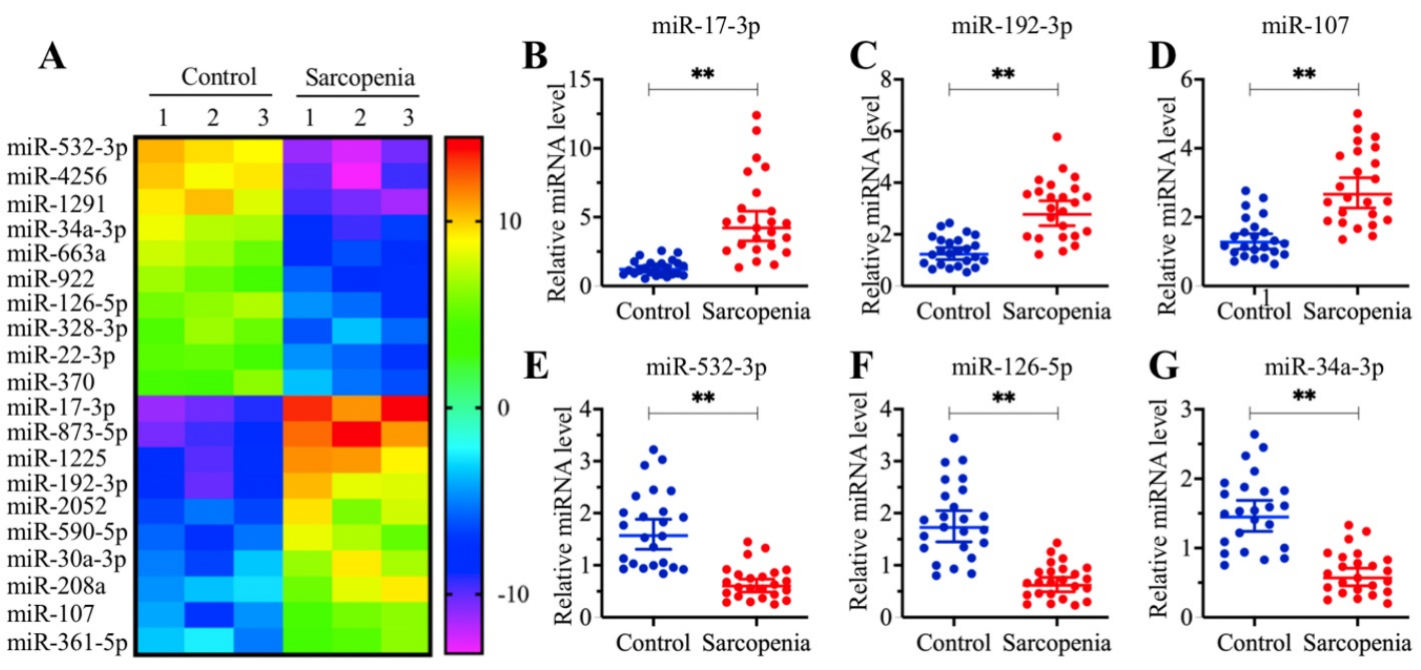
** The expression of miR-532-3p was significantly decreased in sarcopenia patients. (A)** The heat map of 20 miRNAs that were differentially expressed in sarcopenia patient samples. Three-paired muscle tissues from healthy controls and sarcopenia patients were subjected to RNA isolation and subsequent microarray assays. The top 20 miRNAs that were aberrantly expressed were shown. **(B-G)** Verification of three upregulated and three downregulated miRNA levels by qRT-PCR. Twenty-four-paired muscle tissues from healthy controls and sarcopenia patients were used for qRT-PCR analyses to measure the relative expression levels of miR-17-3p **(B)**, miR-192-3p **(C)**, miR-107 **(D)**, miR-532-3p **(E)**, miR-126-5p **(F)**, and miR-34a-3p **(G)**. The expression of individual miRNAs in one healthy control sample was defined as one-fold. ** P < 0.01.

Multiple miRNAs have been shown to play roles in the pathogenesis of sarcopenia 30. To identify aberrantly expressed miRNAs that are associated with human sarcopenia pathogenesis, we conducted a miRNA-based microarray assay comparing three independent sarcopenia muscle tissue samples with three different young adult muscle tissue samples. Overall, we obtained 53 miRNAs that showed aberrant levels in all three sarcopenia muscle tissue samples. Of these, 22 miRNAs were upregulated, while the other 31 miRNAs were downregulated (Supplementary Table 2). In Figure 2A, we showed the 20 miRNAs (10 upregulated and 10 downregulated miRNAs) with the most obvious expression changes. Of these 20 miRNAs, miR-532-3p showed the most significant level of downregulation, while miR-17-3p showed the most obvious level of upregulation (Figure 2A). To examine the reliability of our microarray results, we randomly selected three upregulated (miR-17-3p, miR-192-3p and miR-107) and three downregulated miRNAs (miR-532-3p, miR-126-5p and miR-34a-3p) to verify their expression in 24-paired healthy controls and sarcopenia samples. Consistently, the qRT-PCR results indicated that the expression levels of miR-17-3p, miR-192-3p and miR-107 were significantly increased (Figures 2B-2D), while the expression levels of miR-532-3p, miR-126-5p and miR-34a-3p were dramatically inhibited in the sarcopenia samples compared to those in the controls (Figures 2E-2G). Based on the fact that miRNAs bind to the 3'-UTRs of their targets to cause their downregulation, we focused our study on these downregulated miRNAs to investigate their targets. For this purpose, we primarily aimed to identify the direct target of miR-532-3p. After examining the potential targets of miR-532-3p in a miRNA database (www.mirdb.org), we obtained a total of 568 candidates (Supplementary Table-3). However, we did not have any clues that allowed us to identify the target of miR-532-3p within this large number of candidates.

### The proapoptotic gene *BAK1* was significantly upregulated in sarcopenia samples

**Figure 3:**
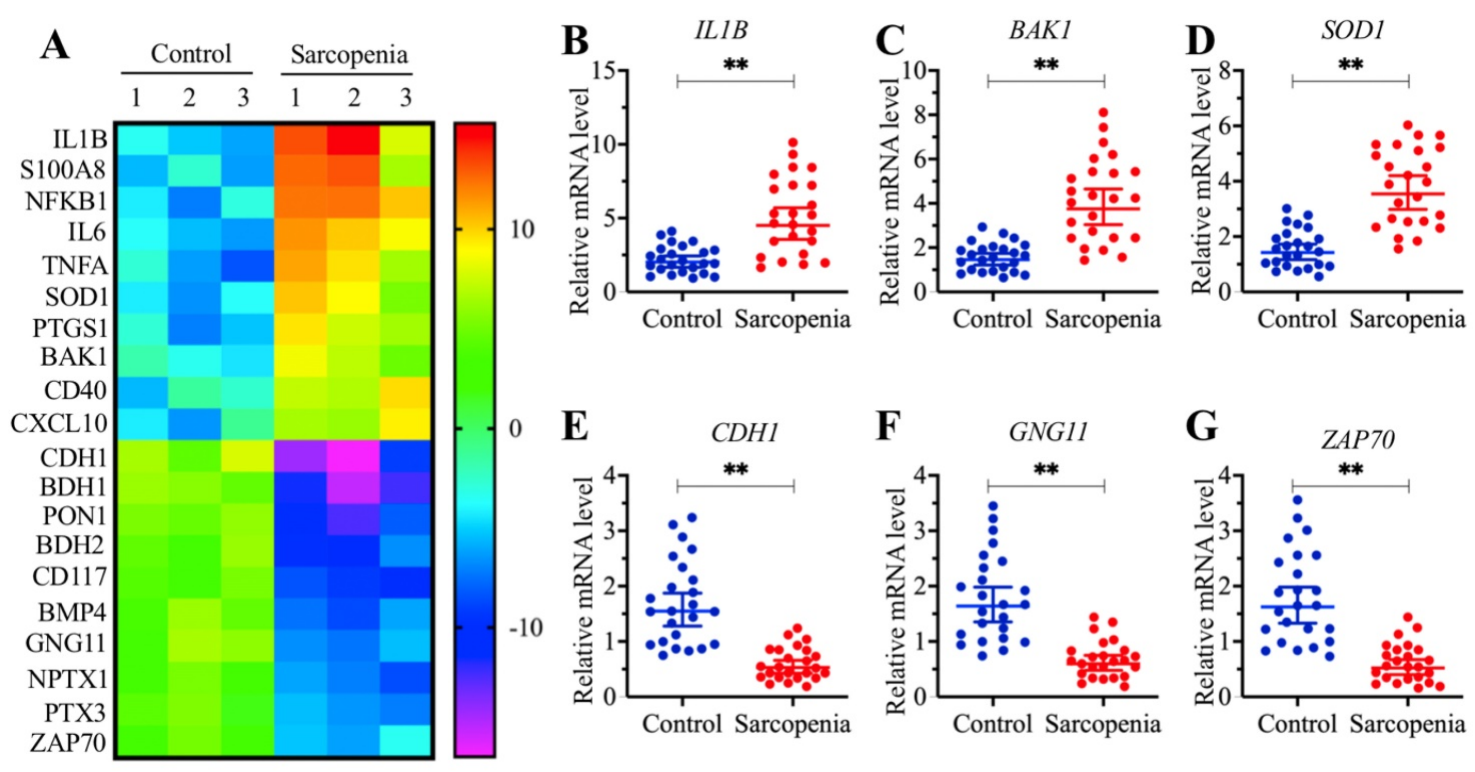
***BAK1* was significantly increased in sarcopenia patients. (A)** The heat map of 20 genes that were differentially expressed in sarcopenia patient samples. Three-paired muscle tissues from healthy controls and sarcopenia patients were subjected to RNA isolation and subsequent microarray assays. The top 20 genes that were aberrantly expressed were shown. **(B-G)** Verification of three upregulated and three downregulated gene levels by qRT-PCR. Twenty-four-paired muscle tissues from healthy controls and sarcopenia patients were used for qRT-PCR analyses to measure the relative expression levels of *IL1B* **(B)**, *BAK1* **(C)**, *SOD1* **(D)**, *CDH1* **(E)**, *GNG11* **(F)**, and *ZAP70* **(G)**. The expression of individual genes in one healthy control sample was defined as one-fold. ** P < 0.01.

To determine the direct target of miR-532-3p that plays a role in the pathogenesis of sarcopenia, we assumed that the downregulation of miR-532-3p in sarcopenia samples would cause the upregulation of its targets. Thus, we conducted a gene-based microarray assay using the same tissue samples as in the miRNA-based microarray assay. Overall, we identified 69 differentially expressed genes in all three sarcopenia muscle tissue samples. Of these, 21 genes were downregulated, while the other 48 genes were upregulated (Supplementary Table-4). As shown in Figure 3A, we showed 20 genes (10 upregulated and 10 downregulated genes) with the most obvious expression changes. To examine the accuracy of our microarray results, we randomly selected three upregulated genes, *IL1B*, *BAK1* and *SOD1* (Superoxide Dismutase 1), and three downregulated genes, *CDH1* (Cadherin 1), *GNG11* (G Protein Subunit Gamma 11) and *ZAP70* (Zeta Chain of T cell Receptor-Associated Protein Kinase 70), to verify their expression in 24-paired healthy controls and sarcopenia samples. We observed that the expression of *IL1B*, *BAK1* and *SOD1* were significantly increased (Figures 3B-3D), while the expression of *CDH1*, *GNG11* and *ZAP70* were dramatically decreased in sarcopenia samples compared to those in the controls (Figures 3E-3G). Next, we compared these 69 differentially expressed genes with the 568 predicted candidate targets of miR-532-3p. Fortunately, we found that a gene known as *BAK1* was included in both gene lists. Thus, we speculated that *BAK1* might be a direct target of miR-532-3p.

### MicroRNA-532-3p directly targeted the 3'-UTR of *BAK1*

**Figure 4:**
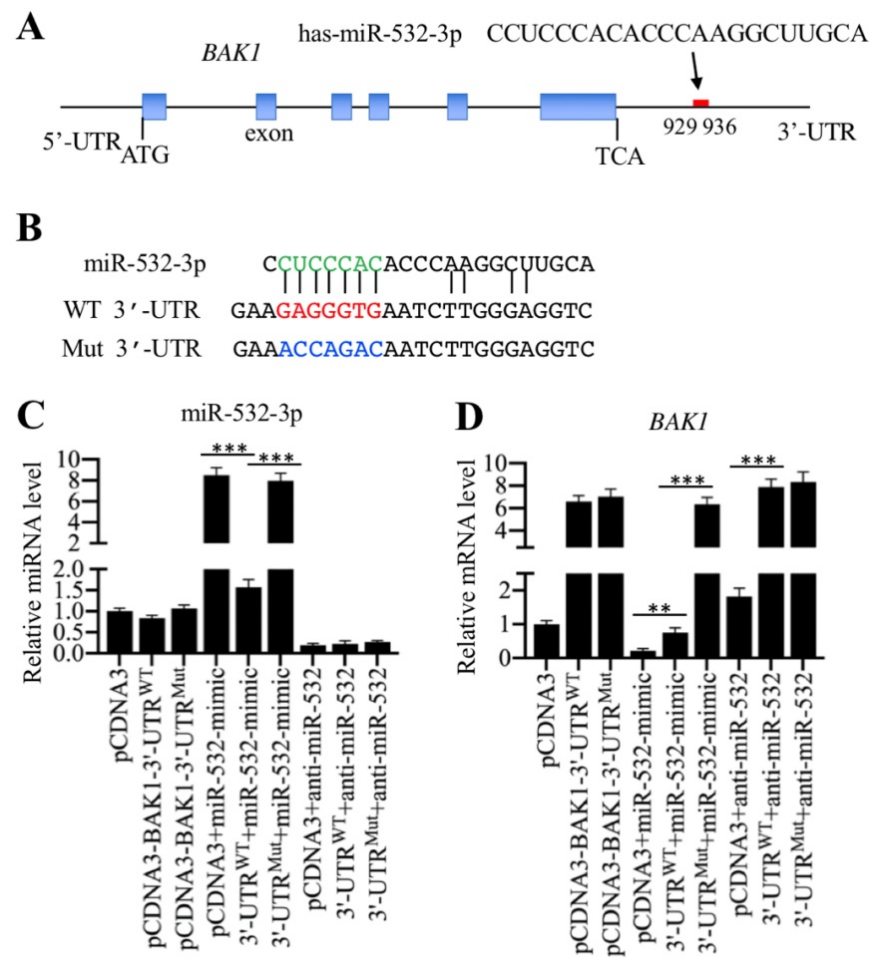
** miR-532-3p specifically bond to the 3′-UTR of *BAK1* and negatively regulated its expression. (A)** Schematic representation of the *BAK1* gene and the binding site of miR-532-3p. The 5′-UTR, exons, introns and 3′-UTR of the *BAK1* gene are shown. Its 3′-UTR has a putative miR-532-3p binding site located between positions 929 and 936 after TCA. **(B)** The wild-type and mutated sequences of *BAK1* 3′-UTR. Two vectors, pCDNA3-BAK1-3′-UTR^WT^ and pCDNA3-BAK1-3′-UTR^Mut,^ were constructed. In the mutant vector, the putative binding site of miR-532-3p was mutated and was indicated by the blue font. **(C and D)** *BAK1* was a direct target of miR-532-3p. The pCDNA3-BAK1-3′-UTR^WT^ and pCDNA3-BAK1-3′-UTR^Mut^ vectors were combined with or without miR-532-3p-mimic and anti-miR-532-3p and transfected into HSMM-1 cells. After incubation for 48 h, cells were harvested and used for RNA isolation, followed by the examination of the expression levels of miR-532-3p **(C)** and *BAK1* **(D)**. ** P < 0.01 and *** P < 0.001.

To verify whether *BAK1* is a direct target of miR-532-3p, we first aimed to determine the potential binding region of miR-532-3p in the 3'-UTR of *BAK1*. In a miRNA target prediction database (www.mirdb.org), we identified a seed sequence (CUCCCAC) in miR-532-3p that could specifically bind to the region from 929-936 in the *BAK1* 3'-UTR (Figure 4A). We then constructed two vectors containing the full length of *BAK1* coding sequence and either the wild type or a mutated 3'-UTR, known as pCDNA3-BAK1-3'-UTR^WT^ and pCDNA3-BAK1-3'-UTR^Mut^. The potential seed sequence of miR-532-3p in the mutant vector was disrupted (Figure 4B). To examine whether the overexpression or the inhibition of miR-532-3p could affect *BAK1* level, we transfected different combinations of vectors, including pCDNA3, pCDNA3-BAK1-3'-UTR^WT^, pCDNA3-BAK1-3'-UTR^Mut^, pCDNA3+miR-532-mimic, pCDNA3-BAK1-3'-UTR^WT^+miR-532-mimic, pCDNA3-BAK1-3'-UTR^Mut^+miR-532-mimic, pCDNA3+anti-miR-532-3p, pCDNA3-BAK1-3'-UTR^WT^+anti-miR-532-3p, and pCDNA3-BAK1-3'-UTR^Mut^+anti-miR-532-3p, into HSMM-1 cells. After confirming that miR-532-3p level was successfully overexpressed or inhibited (Figure 4C), the *BAK1* mRNA level was determined by qRT-PCR analysis. Our results indicated that the overexpression of miR-532-3p could significantly reduce *BAK1* mRNA level in cells expressing pCDNA3-BAK1-3'-UTR^WT^ but not in cells expressing pCDNA3-BAK1-3'-UTR^Mut^ (Figure 4D). In contrast, the inhibition of miR-532-3p significantly increased *BAK1* mRNA level in cells expressing pCDNA3-BAK1-3'-UTR^WT^ or pCDNA3-BAK1-3'-UTR^Mut^ (Figure 4D). These results clearly showed that *BAK1* was a direct target of miR-532-3p and that miR-532-3p bound to its 3'-UTR through the CUCCCAC seed sequence.

### *In vitro* LPS treatment caused inverse effects on miR-532-3p and *BAK1* expression

**Figure 5:**
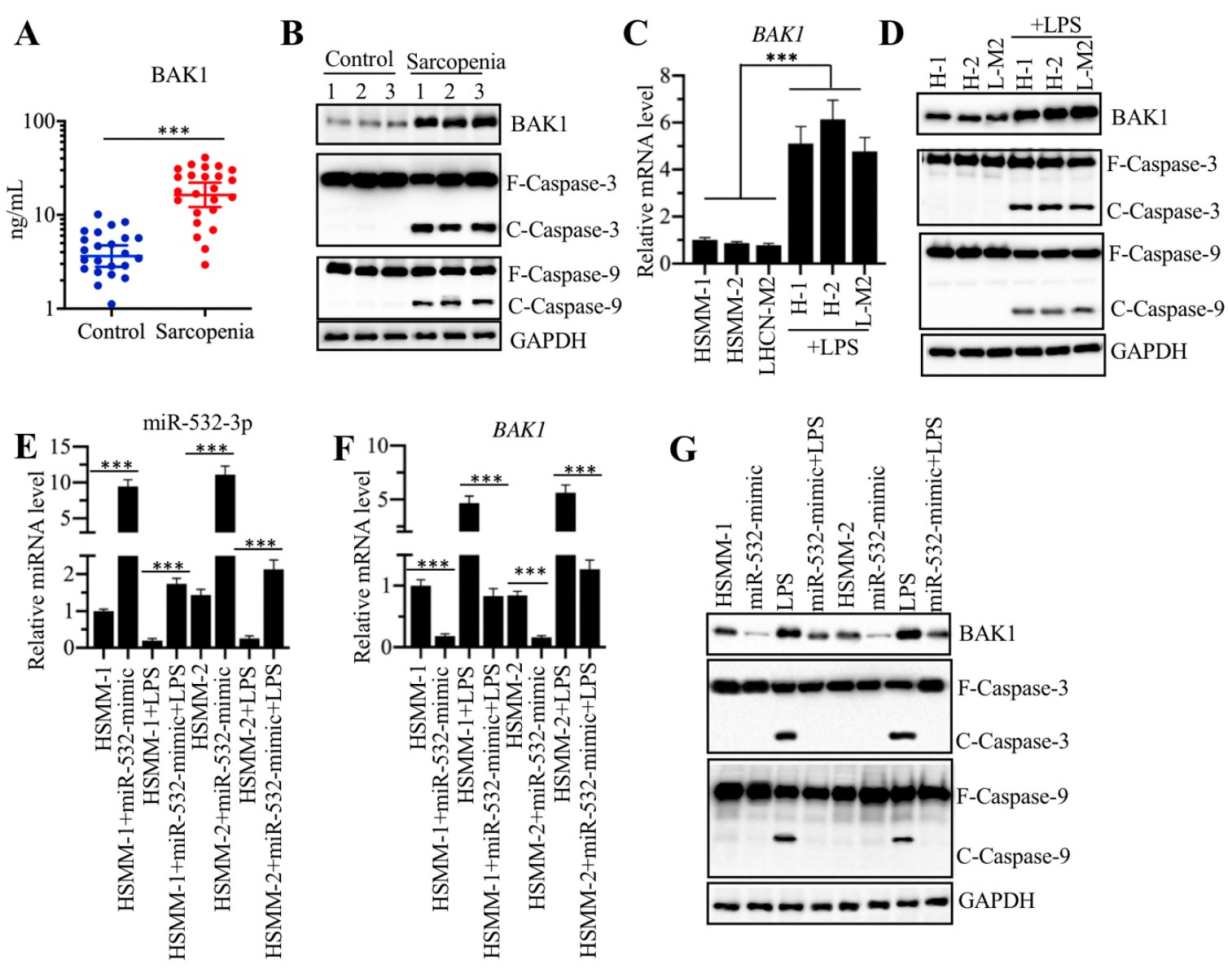
** LPS treatment could affect the expression of miR-532-3p and *BAK1* and their downstream molecules. (A)** Serum levels of BAK1 were significantly elevated. Serum samples from 24 healthy and 24 sarcopenia patients were used to measure circulating BAK1 concentrations using ELISA. *** P < 0.001. **(B)** BAK1 and its downstream caspase cascades were activated in sarcopenia samples. Three-paired muscle tissues from healthy controls and sarcopenia patients were used to detect the protein levels of BAK1, Caspase-3 and Caspase-9. GAPDH was used as a loading control. F: full length; C: cleaved length. **(C)** LPS induced the expression of *BAK1*. Three cell lines, HSMM-1, HSMM-2 and LHCN-M2, were treated with 200 ng/mL LPS for 6 h, followed by RNA isolation and qRT-PCR analyses to measure the mRNA level of *BAK1*. *** P < 0.001. **(D)** LPS induced the BAK1 protein level and its downstream caspase cascades. The cells used in (C) were subjected to protein extraction, followed by the detection of the BAK1, Caspase-3 and Caspase-9 protein levels. GAPDH was used as a loading control. **(E-G)** The combined effects of miR-532-3p-mimic/anti-miR-532-3p and LPS treatment. The HSMM-1 and HSMM-2 cells were primarily transfected with miR-532-3p-mimic or anti-miR-532-3p. After 48 h, cells were treated with 200 ng/mL LPS for 6 h and then were collected and subjected to RNA isolation and protein extraction. The resulting RNA and protein extracts used for the detection of the expression of miR-532-3p **(E)**, *BAK1* mRNA **(F)**, and BAK1 and caspase protein levels **(G)**. *** P < 0.001.

The above results showed that *BAK1* mRNA level was significantly increased in sarcopenia samples (Figure 4C). To determine if its protein level was also increased, we measured serum BAK1 level with an ELISA assay using samples from 24 sarcopenia patients and 24 healthy controls. Our results showed that the circulating BAK1 level in sarcopenia patients was much higher than that in healthy controls (Figure 5A). In addition, we also examined the BAK1 protein levels in three-paired muscle tissues from sarcopenia patients and healthy controls. Our results indicated that BAK1 was significantly increased in the three sarcopenia samples (Figure 5B and Supplementary Figure 2A). Given that BAK1 is a proapoptotic protein and that its induction can result in the activation of downstream caspase cascades, we next sought to determine if apoptotic signaling was activated in the sarcopenia samples. As expected, two caspases, caspase-3 and caspase-9, were activated in all three sarcopenia samples (Figure 5B and Supplementary Figure 2A). The above results also indicated that the sarcopenia serum samples had increased levels of proinflammatory cytokines, and we next sought to determine if the inflammatory status could affect the expression of *BAK1*. For this purpose, we firstly treated HSMM-1 cells with different concentrations of LPS (0, 50, 100 and 200 ng/mL) and IL-1β (0, 1, 5 and 25 ng/mL), followed by determining miR-532-3p and *BAK1* levels. As shown in Supplementary Figure 2B, both LPS and IL-1β treatments caused a gradual downregulation of miR-532-3p but a gradual induction of *BAK1*. Moreover, we also measured three NF-κB target genes including *IL1B*, *IL6* and *TNFA* to indicate the activation of TLR4/NF-κB signaling with the treatments of LPS and IL-1β (Supplementary Figure 2C). After verification of the effectiveness of LPS treatment in our system, we treated three human skeletal muscle myoblast cell lines, HSMM-1, HSMM-2 and LHCN-M2, with 200 ng/mL LPS, followed by evaluating the activation of apoptotic pathway. Consistent with the results in LPS-treated HSMM-1 cells, we also observed the significant induction of *BAK1* mRNA level in the other two cell lines (Figure 5C). The immunoblot results indicated that LPS treatment could increase BAK1 protein level and activate apoptotic pathway (Figure 5D and Supplementary Figure 2D). Since *BAK1* is a direct target of miR-532-3p, it was possible that the inflammatory state caused the downregulation of miR-532-3p prior to *BAK1* upregulation. To verify this possibility, we transfected miR-532-3p-mimic into HSMM-1 and HSMM-2 cells, followed by treatment with or without 200 ng/mL LPS. Our qRT-PCR results indicated that LPS treatment caused the downregulation of miR-532-3p in cells transfected with or without miR-532-3p-mimic compared to that in the corresponding maternal cell lines (Figure 5E). In the same conditions, LPS treatment resulted in inverse effects on *BAK1* mRNA levels (Figure 5F). Moreover, we also evaluated the combined effects of LPS treatment and miR-532-3p overexpression on BAK1 protein level and apoptotic caspases. The results indicated that the overexpression of miR-532-3p alone decreased BAK1 protein level and inactivated Caspase-3 and Caspase-9 (Figure 5G and Supplementary Figure 2E). However, LPS treatment alone could cause the accumulation of BAK1 and activate Caspase-3 and Caspase-9 (Figure 5G and Supplementary Figure 2E). Although LPS treatment could rescue the protein level of BAK1 that was downregulated by miR-532-3p overexpression, it could not activate Caspase-3 and Caspase-9 (Figure 5G and Supplementary Figure 2E). These results suggested that the inflammatory state reduced the miR-532-3p level, which in turn reduced its inhibition of *BAK1* and caused the activation of caspase cascades.

### The NF-κB subunit p50 specifically regulated the expression of miR-532-3p

**Figure 6:**
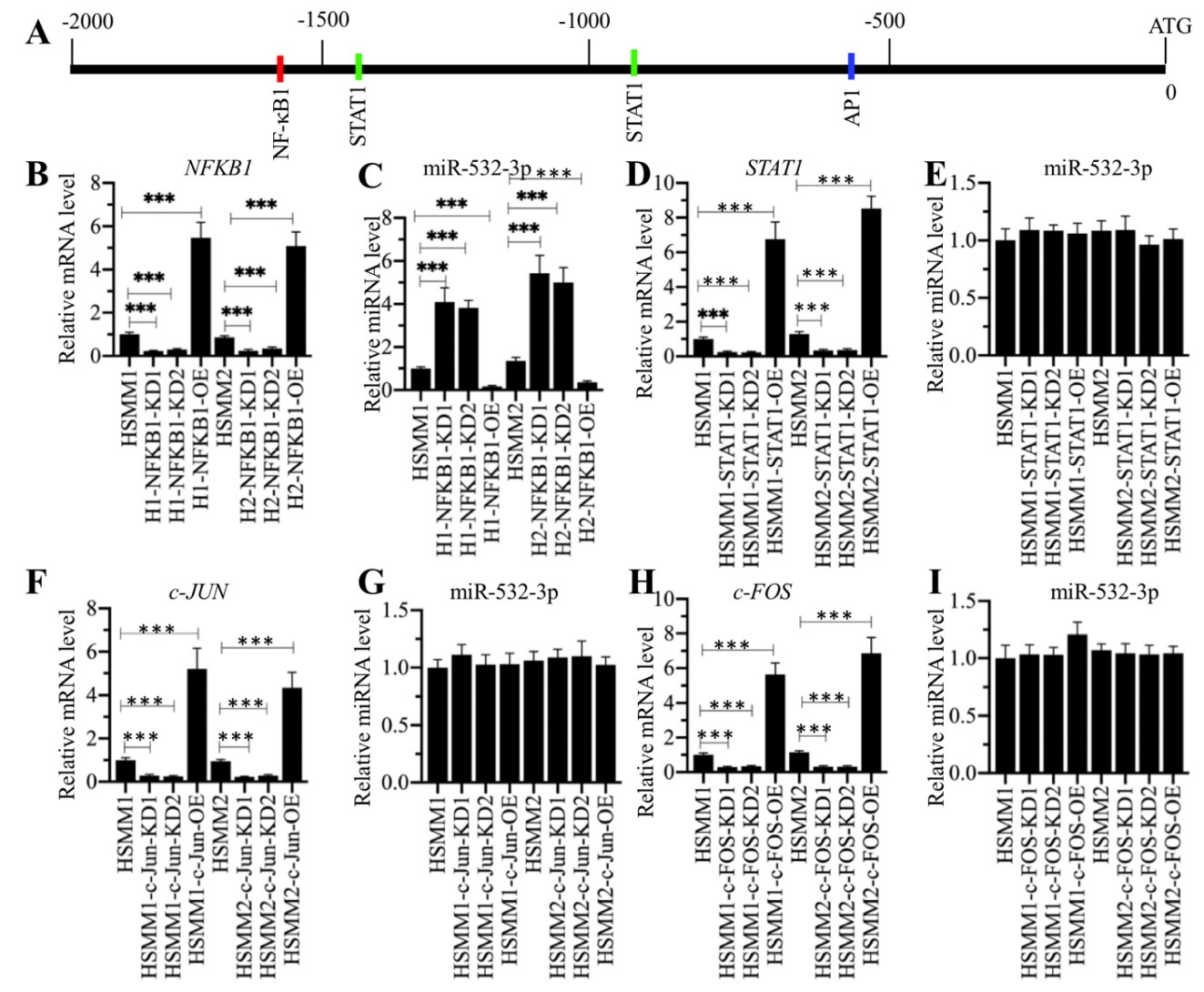
** NFKB1/p50 specifically repressed the expression of miR-532-3p. (A)** The transcription factor binding sites in the promoter of miR-532-3p. A 2000 bp-length promoter fragment was used to predict the transcription factor binding sites. The binding positions of NFKB1 (p50), STAT1 and AP-1 were indicated. **(B-I)** The effects of overexpressing or downregulating different transcription factors on miR-532-3p level. HSMM-1 and HSMM-2 cells were transfected with individual transcription factor-specific siRNAs and the corresponding overexpression vectors to generate the knockdown (KD) and overexpression (OE) cells. The resulting cells were subjected to RNA isolation, followed by the measurement of miR-532-3p level using qRT-PCR analyses. **(B and C)** The expression levels of *NFKB1* **(B)** and miR-532-3p **(C)** in NFKB1-KD and NFKB1-OE cells. *** P < 0.001. **(D and E)** The expression levels of *STAT1* **(D)** and miR-532-3p **(E)** in STAT1-KD and STAT1-OE cells. *** P < 0.001. **(F and G)** The expression levels of *c-JUN* **(F)** and miR-532-3p **(G)** in c-JUN-KD and c-JUN-OE cells. *** P < 0.001. **(H and I)** The expression levels of *c-FOS* **(H)** and miR-532-3p **(I)** in c-FOS-KD and c-FOS-OE cells. *** P < 0.001.

We next sought to determine the mechanism underlying miR-532-3p downregulation. The current understanding regarding the molecular mechanisms of the aberrant expression of miRNA mainly implicates epigenetic modifications (e.g., DNA methylation and acetylation) and transcriptional regulation controlled by transcription factors 35, 36. To explore the possibility of epigenetic modifications, we first checked whether the adjacent region of the miR-532-3p promoter contained a CpG island. Unfortunately, we did not identify a CpG island (Supplementary Figure 3A). We then treated cells with the DNA methylation inhibitor AZA and the histone acetylation activator TSA and evaluated their effects on miR-532-3p level. As shown in Supplementary Figure 3B, neither AZA or TSA treatment changed miR-532-3p level. These results, as well as the fact that that inflammation can activate some transcription factors [e.g., NF-κB and activator protein 1 (AP1)], raised the possibility that the expression of miR-532-3p was controlled by transcription factors. To verify this possibility, we predicted the transcription factor binding sites in a 2000-bp fragment of the promoter of miR-532-3p by using a database (www.alggen.lsi.upc.es). We selected several transcription factors that have been shown to be regulated by inflammation and searched for their binding sites in the promoter of miR-532-3p (Figure 6A). These transcription factor binding sites included one NFKB1 (p50) site, two STAT1 (signal transducer and activator of transcription 1) sites, and one AP1 site. To determine whether these three transcription factors can affect miR-532-3p level, we individually knocked down or overexpressed each of them and further determined their effects on miR-532-3p level. Accordingly, we used two independent siRNAs and one overexpression plasmid of these transcription factors to transfect cells, and then determined the successful transfection by detecting their mRNA and protein levels (Supplementary 4). Our qRT-PCR results indicated that the knockdown or overexpression of *NFKB1* in HSMM-1 and HSMM-2 cells caused the upregulation or downregulation of miR-532-3p, respectively (Figures 6B and 6C). However, we did not observe a significant change in miR-532-3p level in cells knocking down or overexpressing the other two transcription factors and their subunits, including *STAT1*, *c-FOS* and *c-JUN* (Figures 6D-6I). These results suggested that NFKB1 (p50) could negatively regulate the expression of miR-532-3p. In addition, we also evaluated the effects of *NFKB1* downregulation and overexpression on *BAK1* level and its downstream signaling. Our results showed that the downregulation or overexpression of *NFKB1* caused the reduction or induction of *BAK1* and the repression or activation of caspase cascades, respectively (Supplementary Figures 5). These results further supported the conclusion that *BAK1* was a target of miR-532-3p and NFKB1 (p50) negatively regulated miR-532-3p expression.

### p50 specifically bond to the promoter of miR-532-3p

**Figure 7:**
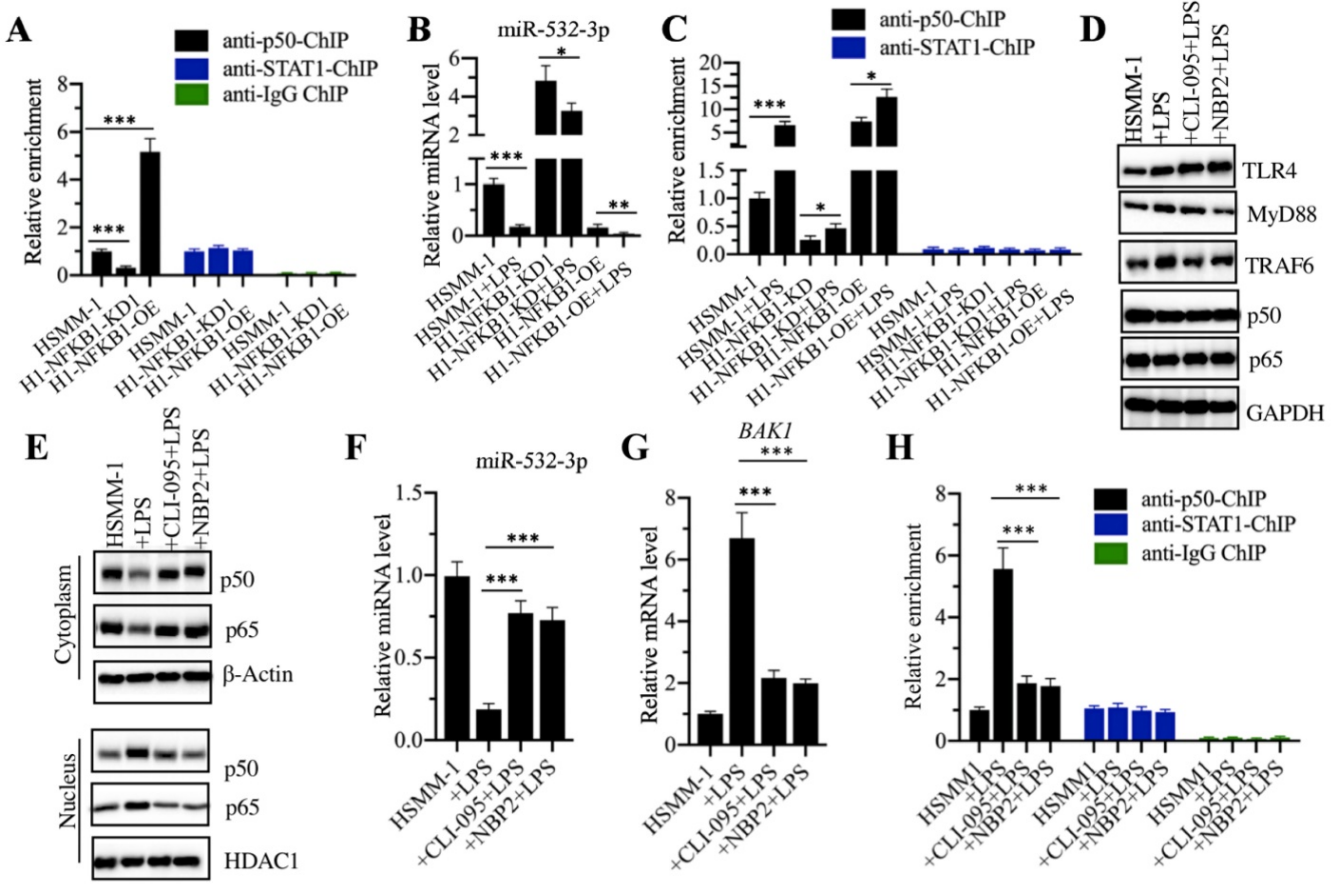
** p50 specifically bond to the promoter of miR-532-3p to repress its expression. (A)** The occupancy of p50 on the promoter of miR-532-3p. The HSMM-1, HSMM1-NFKB1-KD and HSMM1-NFKB1-OE cells were subjected to ChIP assays using anti-p50, anti-STAT1 and IgG antibodies, followed by qRT-PCR analyses to measure the enrichment of p50 on the promoter of miR-532-3p. *** P < 0.001.** (B)** LPS treatment decreased miR-532-3p level. The HSMM-1, HSMM1-NFKB1-KD and HSMM1-NFKB1-OE cells were treated with or without 200 ng/mL LPS for 6 h, followed by RNA isolation and qRT-PCR to measure miR-532-3p level. ** P < 0.01 and *** P < 0.001. (**C)** LPS treatment increased the occupancy of p50 on the promoter of miR-532-3p. The cells used in (B) were subjected to ChIP assays with anti-p50 and anti-STAT1 antibodies, followed by qRT-PCR analyses to measure the enrichment of p50 and STAT1 on the promoter of miR-532-3p. **(D and E)** The blockage of upstream NF-κB signaling prevented the translocation of p50 from the cytoplasm to the nucleus. HSMM-1 cells were primarily treated with the TLR4 inhibitor CLI-095 and the MyD88 inhibitor NBP2-29328 for 6 h, followed by treatment with 200 ng/mL LPS for 6 h. The resulting cells were subjected to total protein extraction and the isolation of cytoplasmic and nuclear protein fractions. The protein levels of TLR4, MyD88, TRAF4, p50 and p65 were determined by immunoblot analyses. GAPDH, β-actin and HDAC1 were used as loading controls. **(F and G)** The blockage of upstream NF-κB signaling blocked the effect of LPS on miR-532-3p expression. The cells used in (D) were used for RNA isolation, followed by qRT-PCR analyses to examine the expression of miR-532-3p **(F)** and *BAK1* **(G)**. **(I)** The blockage of upstream NF-κB signaling decreased the occupancy of p50 on the promoter of miR-532-3p. The cells used in (D) were used for ChIP assays with anti-p50, anti-STAT1 and IgG antibodies, followed by qRT-PCR analyses to measure the enrichment of p50 and STAT1 on the promoter of miR-532-3p. *** P < 0.001.

To determine how p50 regulates miR-532-3p expression, we performed a ChIP assay using anti-p50 and anti-STAT1 in HSMM-1, HSMM-1-NFKB1-KD and HSMM-1-NFKB1-OE cells. Our results showed that the occupancy of p50 on the promoter of miR-532-3p was dramatically decreased by *NFKB1* knockdown but significantly increased by *NFKB1* overexpression (Figure 7A). In contrast, we also detected the enrichment of STAT1 in the same cells, but we did not find a change in the presence of *NFKB1* knockdown and overexpression. Next, we evaluated the combined effects of LPS and *NFKB1* knockdown or overexpression on the expression of miR-532-3p. Accordingly, we treated HSMM-1, HSMM-1-NFKB1-KD and HSMM-1-NFKB1-OE cells with or without LPS. Our results indicated that LPS treatment dramatically decreased the expression of miR-532-3p in all three cell backgrounds compared to that in the corresponding untreated cells (Figure 7B). Using these cells, we also evaluated the occupancy of p50 on the promoter of miR-532-3p. The qRT-PCR results indicated that LPS treatment enhanced the enrichment of p50 on the promoter of miR-532-3p (Figure 7C), while no significant change was observed in ChIP samples immunoprecipitated with anti-STAT1 (Figure 7C). Our results showed that the activation of the TLR4/NF-κB signaling axis mediated the expression of miR-532-3p. We next sought to block this signaling using the TLR4 inhibitor CLI-095 and the MyD88 inhibitor NBP2-29328, followed by the evaluation of the occupancy of p50 on the promoter of miR-532-3p. Our results indicated that CLI-095 and NBP2-29328 significantly inhibited the translocation of p50 and p65 from the cytoplasm to the nucleus following LPS treatment (Figures 7D and 7E). Meanwhile, the expression of miR-532-3p and *BAK1* were significantly upregulated and decreased, respectively (Figures 7F and 7G). The ChIP assay results showed that the occupancy of p50 was significantly downregulated when cells were treated with LPS (Figure 7I). Collectively, these results suggested that TLR4-mediated signaling activated p50, which specifically bound to the promoter of miR-532-3p and negatively regulated its expression.

## Discussion

**Figure 8:**
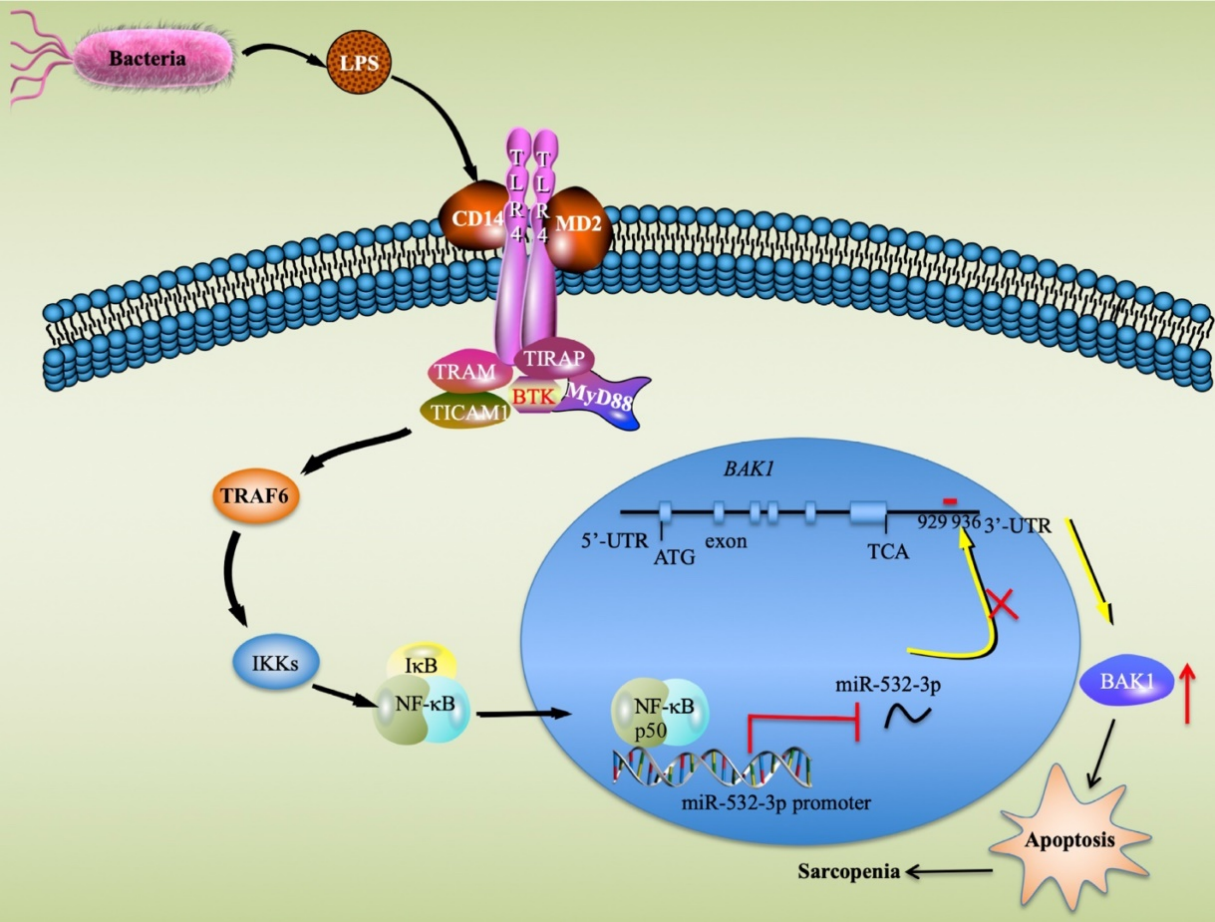
** A schematic model of the upstream and downstream signaling pathways of miR-532-3p in sarcopenia.** LPS secreted from bacteria activates the TLR4/NF-κB signaling axis and causes the translocation of the NF-κB transcription factor from the cytoplasm to the nucleus, where the p50 subunit specifically binds to the promoter of miR-532-3p and inhibits its expression. The decrease in miR-532-3p results in the upregulation of *BAK1*, which activates its downstream apoptotic signaling and causes the activation of caspase cascades, eventually leading to apoptosis and the occurrence of sarcopenia.

Sarcopenia is a degenerative disorder associated with the aging process 3. Although inflammation and apoptosis are considered to be two major causes that contribute to the pathogenesis of sarcopenia 1, 2, it is still unclear which signaling pathways and critical molecules are involved in this process. In the present study, we identified multiple miRNAs and genes that were aberrantly expressed in sarcopenia patient samples, which provides valuable information for future studies of sarcopenia. Molecularly, our results support a model in which LPS secreted by gram-negative bacteria binds to the CD14-TLR4-MD2 complex and activates TLR4 downstream signaling. The activation of the TLR4/NF-κB signaling axis results in the translocation of NF-κB subunits from the cytoplasm to the nucleus, where the p50 subunit specifically binds to the promoter of miR-532-3p and thus represses its expression. The downregulation of miR-532-3p abolishes its inhibition of the proapoptotic gene *BAK1*, causing the activation of BAK1-dependent downstream caspase cascades and eventually leading to apoptosis and the occurrence of sarcopenia (Figure 8).

In recent decades, miRNAs have emerged as important factors that contribute to different diseases 23-25. Similarly, researchers have also identified multiple miRNAs that may play roles in the pathogenesis of sarcopenia process 30. However, the majority of these miRNAs are derived from mice, and thus we know little about the roles of human-specific miRNAs in the pathological development of sarcopenia process 30. To solve this problem, we conducted a microarray analysis using human sarcopenia samples. Here, we clearly revealed the upstream and downstream signaling of miR-532-3p, which will greatly enhance our understanding of how inflammation and apoptosis coordinate and contribute to the occurrence of diseases. Recently, miR-532-3p has also been shown to play roles in other biological processes, especially in tumorigenesis. For instance, the overexpression of miR-532-5p can improve the prognosis of ovarian cancer through targeting *CSNK2A2 (Casein Kinase 2 Alpha 2), CHD4 (Chromodomain Helicase DNA Binding Protein 4)*, and *SH3PXD2A* (SH3 and PX Domain-containing Protein 2A) 37. The miR-532-3p level can be used to predict the efficiency of rituximab-mediated lymphodepletion in chronic lymphocytic leukaemia (CLL) patients because of its effect on the expression of CD20 family members 38. In addition, miR-532-5p also exhibits antiviral activity against West Nile Virus via the suppression of the host genes *SESTD1* (SEC14 and Spectrin Domain-Containing 1) and *TAB3* (TGF-beta-activated Kinase 1 Binding Protein 3) 39. Besides miR-532-3p, we also found other miRNAs that showed significant decreases in expression (Supplementary Table-2). After predicting the potential targets of these differentially expressed miRNAs, we found that some of them targeted proinflammatory cytokine genes, which were also aberrantly expressed according to the gene-based microarray results. For instance, miR-150-5p, miR-495-3p and miR-1323 were significantly downregulated according to the miRNA-based microarray results, and they were predicted to target *TNFA*, *IL1B*, and *IL-6*, respectively (Supplementary Figure 6). These results suggest that our microarray results are reliable. Most importantly, the results imply that the pathological process underlying sarcopenia may result from the combined effects and regulation of different miRNAs and genes, not only miR-532-3p and its target *BAK1*. We are currently investigating the individual roles and contributions of these factors to the pathogenesis of sarcopenia.

In the present study, we also revealed the mechanism underlying miR-532-3p downregulation in inflammatory conditions. By excluding the possibility of epigenetic modification, we found that the overexpression or downregulation of *NFKB1* can cause inverse effects on the expression level of miR-532-3p. With additional ChIP assays, we demonstrated that p50 specifically bond to the promoter region of miR-532-3p and negatively regulated its expression. The current understanding of the molecular mechanisms involved in miRNA dysregulation mainly implicates epigenetic and transcriptional regulation. Here, we found that the transcription factor p50 negatively controlled miR-532-3p expression. To the best of our knowledge, only a few studies have revealed that transcription factors can control miRNA expression. For example, Fendler and colleagues showed that HNF-1β (Hepatocyte Nuclear Factor 1-beta) was capable of controlling the expression of miR-24, miR-27b, miR-199a, and miR-223 in diabetic patients 40. Mitxelena and colleagues revealed that E2F7 was involved in the regulation of miR-25, miR-26a, miR-27b and miR-92a 41. Transcription factors often form complexes with histone modification enzymes (e.g., histone acetyltransferase and deacetylases), corepressors and coactivators to control gene 28, 42. It is still unknown whether p50 can form a transcriptional complex with other proteins to regulate miR-532-3p expression. We are currently performing immunoprecipitation assays using NFKB1 as bait to attempt to identify how NFKB1 negatively controls miR-532-3p expression.

In summary, we found that the activation of the TLR4/NF-κB signaling axis causes the cytoplasmic- nuclear translocation of p50, which binds to the promoter of miR-532-3p and represses its expression. The decreased expression of miR-532-3p eliminates its inhibition of *BAK1* and causes the latter to be upregulated, which further contributes to the activation of downstream apoptotic signaling.

## Supplementary Material

Supplementary figures and tables.
