# Supplementary material for: Inflammation-dependent downregulation of miR-532-3p mediates apoptotic signaling in human sarcopenia through targeting BAK1
Source: Int J Biol Sci. 2020 Feb 24;16(9):1481–94. doi: 10.7150/ijbs.41641 (PMC7097925; doi:10.7150/ijbs.41641)
Supplement: Supplementary file 1 — Supplementary figures and tables. [file ijbsv16p1481s1.pdf]

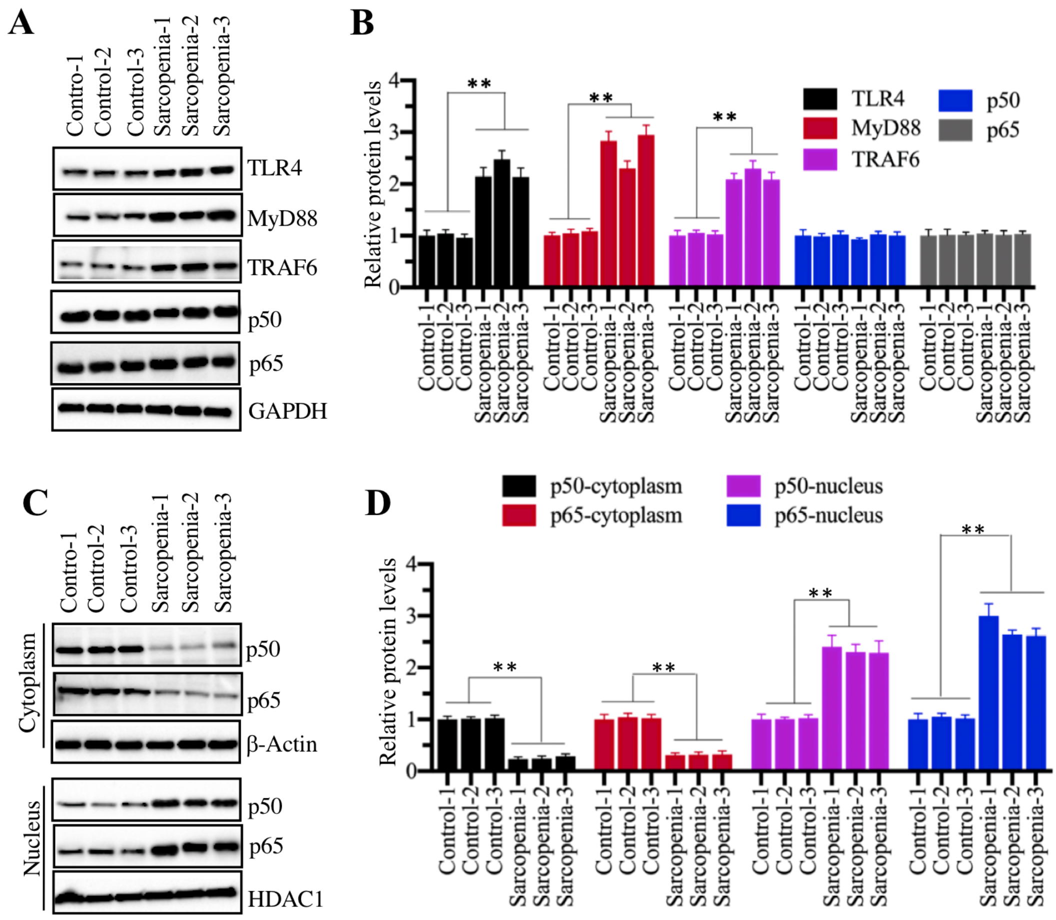

Supplementary Figure 1. The TLR4/NF-κB signalling axis was activated in sarcopenia patient samples

Three-paired muscle tissues from healthy controls and sarcopenia patients were subjected to total protein extraction and isolation of the cytoplasmic and nuclear protein fractions. **(A** and **B)** The protein levels of TLR4, MyD88, TRAF4, p50 and p65 in total cell extracts. Total cell extracts were subjected to immunoblot analyses to determine protein levels of TLR4, MyD88, TRAF4, p50 and p65. GAPDH was used as a loading control **(A)**. **(B)** The quantified protein levels in (A). ** $P < 0.01$. **(C** and **D)** The protein levels of p50 and p65 in the cytoplasmic and nuclear protein fractions. The purified cytoplasmic and nuclear fractions were subjected to immunoblot analyses to determine

protein levels of p50 and p65. β-actin and HDAC1 were used as loading controls. **(D)** The quantified protein levels in (C). ** $P < 0.01$.

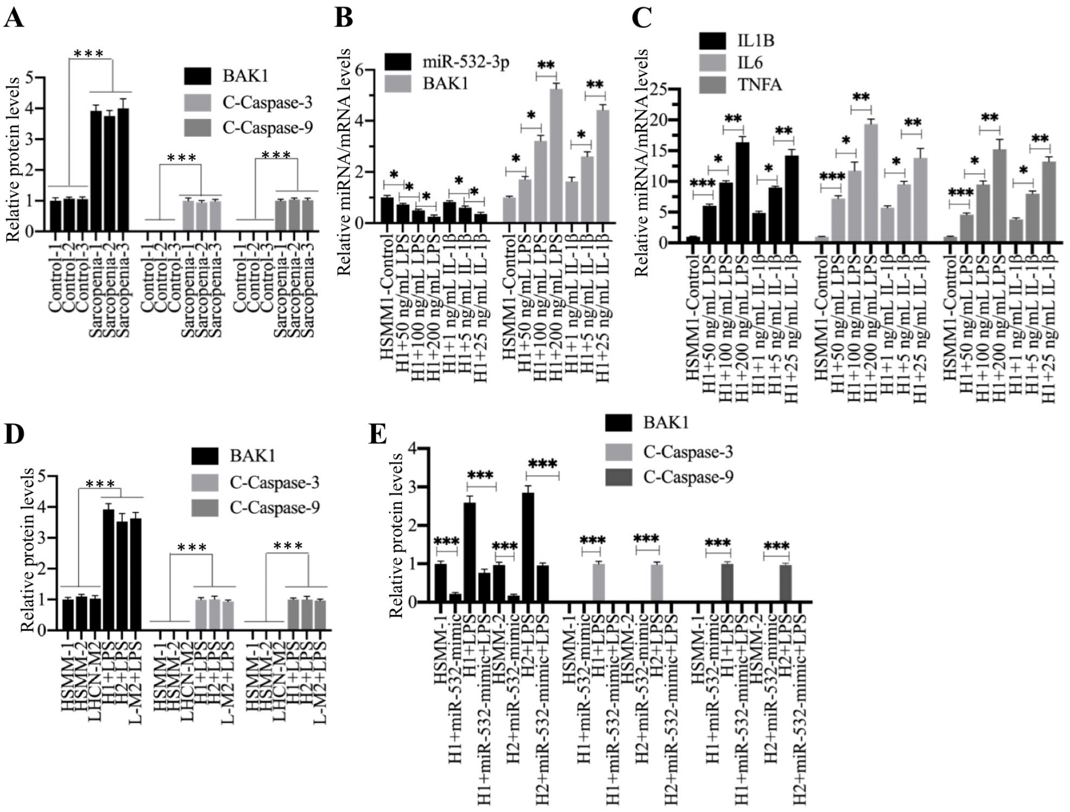

Supplementary Figure 2. The effects of LPS and IL-1β on miR-532-3p and its downstream molecules

(A) The relative protein levels of BAK1 and Caspases in sarcopenia samples. The protein signals in Figure 5B were quantified using Image J software. The protein levels of BAK1 in Control-1 was defined as one-fold. The protein levels of C-Caspase3 and C-caspase-9 in sarcopenia-1 were defined as one-fold. *** $P < 0.001$. **(B)** The effects of different concentrations of LPS and IL-1β on the expression of miR-532-3p and *BAK1*. The HSMM-1 cells were treated with different concentrations of LPS (0, 50, 100 and 200 ng/mL) and IL-1β (0, 1, 5 and 25 ng/mL), followed by qRT-PCR analyses to examine the expression of miR-532-3p and *BAK1*. * $P < 0.05$ and ** $P < 0.01$. **(C)** The effects of different concentrations of LPS and IL-1β on the expression of NF-κB target genes. The cDNA samples used in (B) were subjected to measure the mRNA levels of

IL1B, *IL6* and *TNFA*. * $P < 0.05$, ** $P < 0.01$ and *** $P < 0.001$. **(D)** The relative protein levels of BAK1 protein level and Caspases in LPS-treated cells. The protein signals in Figure 5D were quantified using Image J software. The protein levels of BAK1 in HSMM-1 was defined as one-fold. The protein levels of C-Caspase3 and C-caspase-9 in LPS-treated HSMM-1 cells were defined as one-fold. *** $P < 0.001$. **(E)** The relative protein levels of BAK1 protein level and Caspases. The protein signals in Figure 5G were quantified using Image J software. The protein levels of BAK1 in HSMM-1 was defined as one-fold. The protein levels of C-Caspase3 and C-caspase-9 in LPS-treated HSMM-1 cells were defined as one-fold. *** $P < 0.001$.

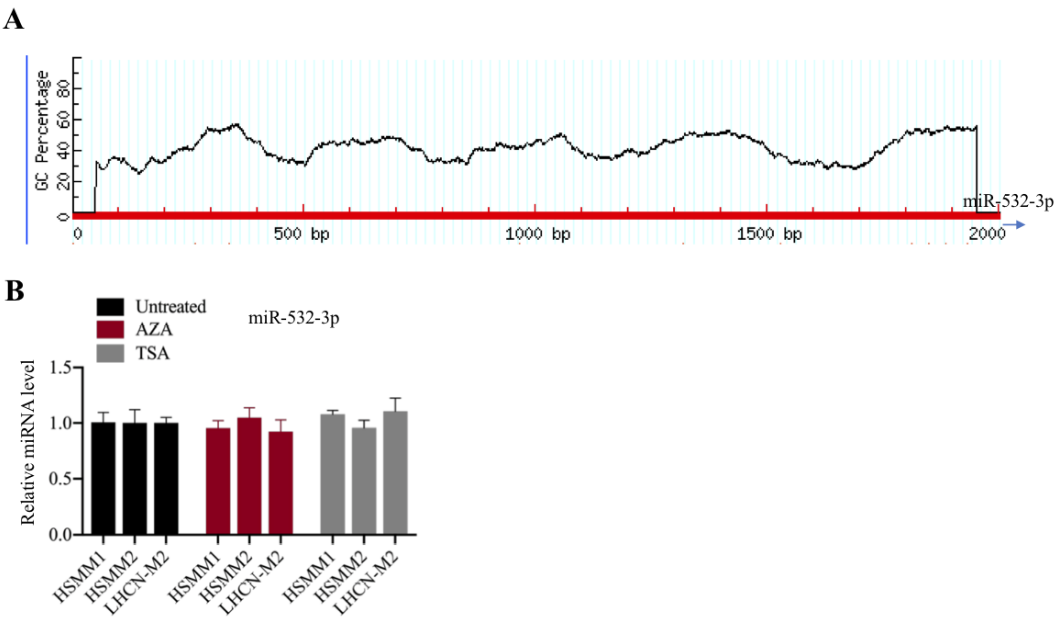

Supplementary Figure 3. Changes in epigenetic modification did not affect miR-532-3p expression

(A) The promoter region of miR-532-3p does not contain a CpG island. A 2000 bp-length promoter fragment from miR-532-3p was used to predict the presence of a CpG island. The position of miR-532-3p is indicated. **(B)** Treatment with the DNA methylation inhibitor AZA and the acetylation activator TSA did not change the miR-532-3p level. Three cell lines, HSMM-1, HSMM-2 and LHCN-M2, were treated with AZA and TSA, followed by qRT-PCR analyses to examine the expression of miR-532-3p.

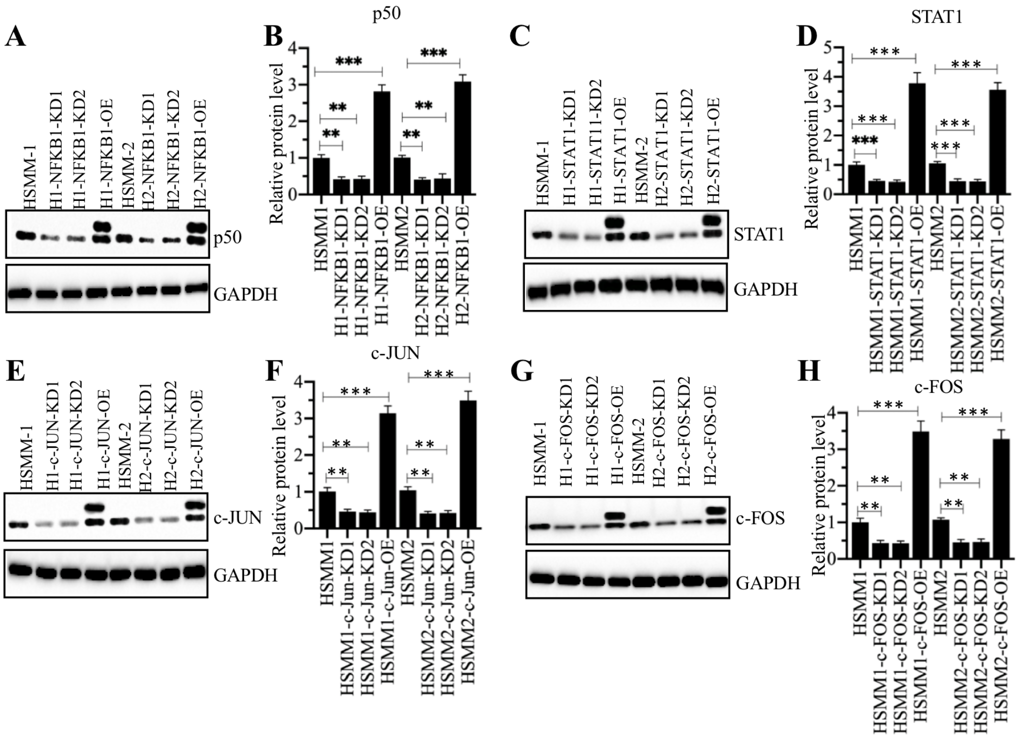

Supplementary Figure 4. The protein levels of transcription factors in their corresponding knockdown and overexpression cells

(A and **B)** The protein levels of p50. Total cell extracts from HSMM-1, HSMM1-NFKB1-KD1, HSMM1-NFKB1-KD2, HSMM1-NFKB1-OE, HSMM-2, HSMM2-NFKB1-KD1, HSMM2-NFKB1-KD2, and HSMM2-NFKB1-OE were subjected to immunoblots to examine the protein level of p50 **(A)**. GAPDH was used as a loading control. **(B)** The protein signals were quantified using Image J software. The protein levels of p50 in HSMM-1 was defined as one-fold. ** $P < 0.01$ and *** $P < 0.001$. **(C** and **D)** The protein levels of STAT1. Total cell extracts from HSMM-1, HSMM1-STAT1-KD1, HSMM1-STAT1-KD2, HSMM1-STAT1-OE, HSMM-2, HSMM2-STAT1-KD1, HSMM2-STAT1-KD2, and HSMM2-STAT1-OE were subjected to immunoblots to examine the protein level of STAT1 **(C)**. GAPDH was

used as a loading control. **(D)** The protein signals were quantified using Image J software. The protein levels of STAT1 in HSMM-1 was defined as one-fold. *** $P < 0.001$. **(E** and **F)** The protein levels of c-JUN. Total cell extracts from HSMM-1, HSMM1-c-JUN-KD1, HSMM1-c-JUN-KD2, HSMM1-c-JUN-OE, HSMM-2, HSMM2-c-JUN-KD1, HSMM2-c-JUN-KD2, and HSMM2-c-JUN-OE were subjected to immunoblots to examine the protein level of c-JUN **(E)**. GAPDH was used as a loading control. **(F)** The protein signals were quantified using Image J software. The protein levels of c-JUN in HSMM-1 was defined as one-fold. *** $P < 0.001$. **(G** and **H)** The protein levels of c-FOS. Total cell extracts from HSMM-1, HSMM1-c-FOS-KD1, HSMM1-c-FOS-KD2, HSMM1-c-FOS-OE, HSMM-2, HSMM2-c-FOS-KD1, HSMM2-c-FOS-KD2, and HSMM2-c-FOS-OE were subjected to immunoblots to examine the protein level of c-FOS **(G)**. GAPDH was used as a loading control. **(H)** The protein signals were quantified using Image J software. The protein levels of c-FOS in HSMM-1 was defined as one-fold. *** $P < 0.001$.

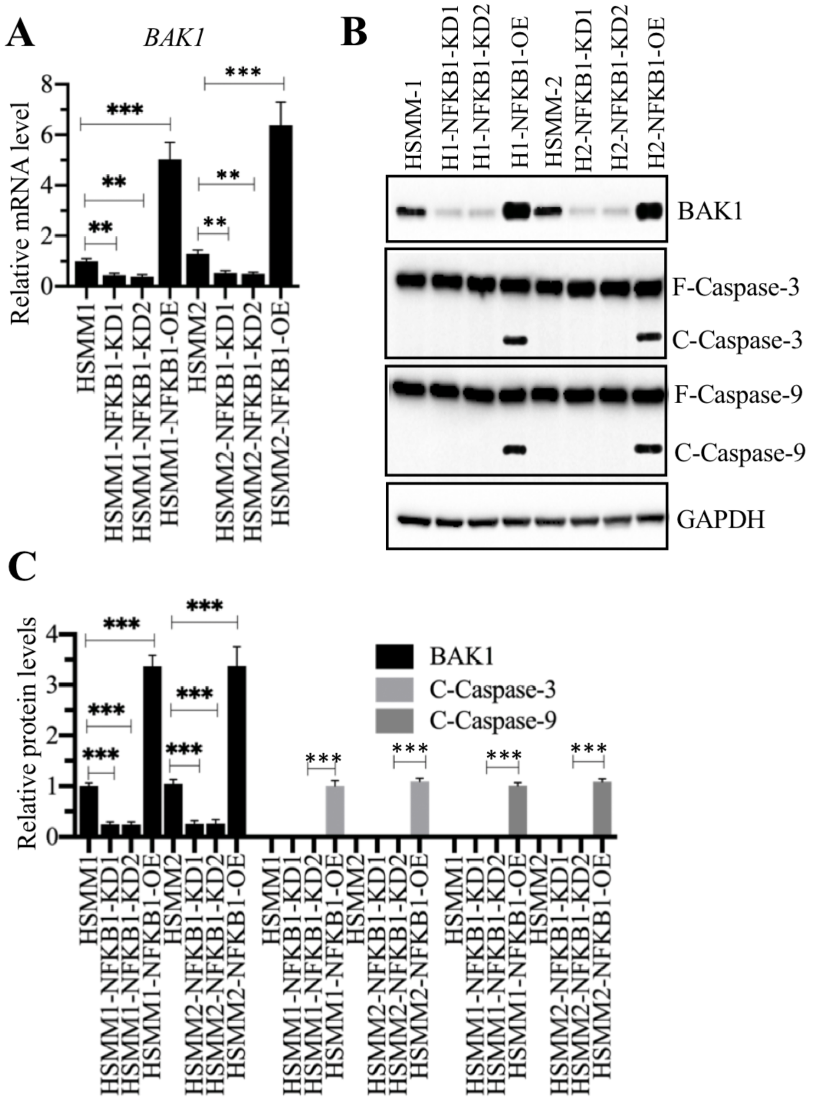

Supplementary Figure 5. Overexpression of *NFKB1* caused the activation of the downstream BAK1 signalling pathway

(A) Overexpression of *NFKB1* increased *BAK1* mRNA levels. The HSMM-1, HSMM1-NFKB1-KD1, HSMM1-NFKB1-KD2, HSMM1-NFKB1-OE, HSMM-2, HSMM2-NFKB1-KD1, HSMM2-NFKB1-KD2 and HSMM2-NFKB1-OE cells were used for RNA isolation, followed by qRT-PCR analyses to examine the expression of *BAK1*. *** $P < 0.001$. **(B** and **C)** Overexpression of *NFKB1* caused the activation of BAK1 downstream signalling pathway. The cells used in (A) were used for protein

extraction. The protein was then used to detect the protein levels of BAK1, Caspase-3 and Caspase-9 **(B)**. GAPDH was used as a loading control. F: full length; C: cleaved length. The protein signals in (B) were quantified using Image J software **(C)**. *** $P < 0.001$.

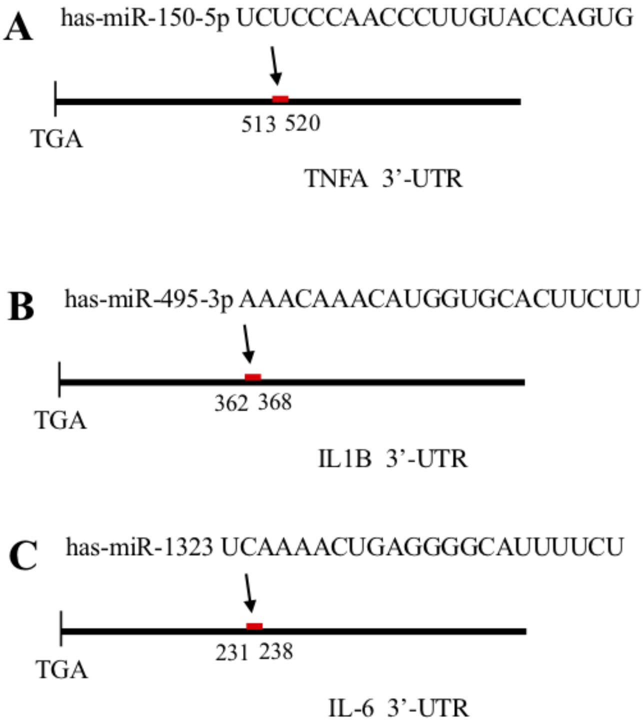

Supplementary Figure 6. Several miRNAs have the potential to target proinflammatory cytokines

Three differentially expressed miRNAs, miR-150-5p, miR-495-3p and miR-1323, have the potential to target *TNFA*, *IL1B* and *IL6*, respectively. The putative binding sites are indicated with arrows.

Supplementary Table-1. Primers used for qRT-PCR analyses

Gene	Forward	Reverse
IL1B	5'-TGTACCTGTCCTGCGTGTT-3'	5'- CTCCCAGGAAGACGGGCATG-3'
BAK1	5'-TGGGCTTCGGCTACCGTCTGGC-3'	5'- GTTCAGGATGGGACCATTGC-3'
SOD1	5'-AGATGGTGTGGCCGATGTG-3'	5'TGTACTTTCTTCATTTCCA-3'
CDH1	5'-CACCACTGGGCTGGACCGAG-3'	5'-TGGGATTGAAGATCGGAGGA-3'
GNG11	5'-CGCTGCCAGAGCTAGCCCGAG-3'	5'- GATCCTCTCCAGAACGTTCTTC-3'
ZAP70	5'-GAGGTCATGGCCTTCATCGA-3'	5'- GCTCCACGGTCAGGAAGTCGG-3'
NFKB1	5'- TAGCTTCCCACACTATGGAT-3'	5'- ACAACCTTCAGGGTCCT-3'
STAT1	5'-TCCTGTCACAGCTGGATGA-3'	5'- CATAGACATCTGGATTG-3'
c-Jun	5'-GCCTTCGTTAACTGTGTAT-3'	5'- AACACTGGGCAGGATACCC-3'
c-FOS	5'-ACTCCAGGGCTGGCGTT-3'	5'-CTTGGAGTGTATCAGTCAGC-3'
β-Actin	5'-AGAGCTACGAGCTGCCTGAC-3	5'- AGCACTGTGTTGGCGTACAG -3'

Supplementary Table-2. Differentially expressed miRNAs in sarcopenia samples

miRNA	Average fold change	P Value	Expression
miR-17-3p	19.3	0.00036	Up
miR-873-5p	16.6	0.00097	Up
miR-1225	15.2	0.00044	Up
miR-192-3p	14.4	0.00067	Up
miR-2052	13.7	0.00024	Up
miR-590-5p	11.6	0.00045	Up
miR-30a-3p	11.2	0.00033	Up
miR-208a	10.9	0.00091	Up
miR-107	10.2	0.00027	Up
miR-361-5p	9.7	0.00046	Up
miR-33a-3p	9.3	0.00082	Up
miR-449c	8.9	0.00012	Up
miR-369	8.4	0.00044	Up
miR-559	7.8	0.00018	Up
miR-572	7.1	0.0024	Up
miR-675	5.9	0.00058	Up
miR-744	5.6	0.00052	Up
miR-1537	4.9	0.00011	Up
miR-1277	4.5	0.00046	Up
miR-382	4.1	0.00082	Up
miR-199a-1	3.4	0.00084	Up
miR-92a-1	2.5	0.00015	Up
miR-532-3p	-18.6	0.00065	Down
miR-4256	-18.1	0.00047	Down
miR-1291	-16.8	0.00024	Down
miR-34a-3p	-15.5	0.00083	Down
miR-663a	-14.3	0.00049	Down
miR-922	-13.2	0.00092	Down
miR-126-5p	-11.9	0.00018	Down
miR-328-3p	-10.6	0.00052	Down

miR-22-3p	-10.3	0.00035	Down
miR-370	-9.5	0.00057	Down
miR-23a	-7.3	0.00096	Down
miR-100	-7.1	0.00013	Down
miR-182	-6.9	0.00025	Down
miR-216a	-6.8	0.00029	Down
miR-150-5p	-6.6	0.00051	Down
miR-495-3p	-6.6	0.00037	Down
miR-1323	-6.4	0.00063	Down
miR-890	-6.2	0.00074	Down
miR-769	-6.1	0.00083	Down
miR-944	-5.8	0.0058	Down
miR-1469	-5.6	0.00052	Down
miR-630	-5.5	0.00064	Down
miR-544a	-5.2	0.00038	Down
miR-377	-5.0	0.00082	Down
miR-640	-4.8	0.00073	Down
miR-622	-4.7	0.00014	Down
miR-2110	-4.2	0.00017	Down
miR-1827	-3.9	0.00026	Down
miR-3168	-3.6	0.00057	Down
miR-4310	-3.3	0.00036	Down
miR-760	-2.6	0.00083	Down

Supplementary Table-3. The predicted targets of miR-532-3p

Target Rank	Target Score	Gene Symbol	Gene Description
**1**	99	MYB	MYB proto-oncogene, transcription factor
**2**	99	CYTH1	cytohesin 1
**3**	98	KLHL12	kelch like family member 12
**4**	98	ZFP90	ZFP90 zinc finger protein
**5**	96	DCAF6	DDB1 and CUL4 associated factor 6
**6**	96	FAM49A	family with sequence similarity 49 member A
**7**	95	HNRNPH3	heterogeneous nuclear ribonucleoprotein H3
**8**	95	BCL9	BCL9, transcription coactivator
**9**	95	ANKRD12	ankyrin repeat domain 12
**10**	94	C22orf46	chromosome 22 open reading frame 46
**11**	94	GALNT10	polypeptide N-acetylgalactosaminyltransferase 10
**12**	93	DDOST	dolichyl-diphosphooligosaccharide--protein glycosyltransferase non-catalytic subunit
**13**	93	CHD3	chromodomain helicase DNA binding protein 3
**14**	93	SZRD1	SUZ RNA binding domain containing 1
**15**	92	TNRC6B	trinucleotide repeat containing 6B
**16**	92	HNRNPH2	heterogeneous nuclear ribonucleoprotein H2
**17**	92	PRDM16	PR/SET domain 16
**18**	91	ZNF543	zinc finger protein 543
**19**	91	TET3	tet methylcytosine dioxygenase 3
**20**	91	WDR36	WD repeat domain 36
**21**	91	ZFP91	ZFP91 zinc finger protein
**22**	90	NIPBL	NIPBL, cohesin loading factor
**23**	90	ALCAM	activated leukocyte cell adhesion molecule
**24**	90	PAPPA	pappalysin 1
**25**	90	PRKCA	protein kinase C alpha
**26**	89	NFASC	Neurofascin
**27**	89	DTX4	deltex E3 ubiquitin ligase 4
**28**	89	GSPT1	G1 to S phase transition 1

**29**	89	QKI	QKI, KH domain containing RNA binding
**30**	89	GALNT6	polypeptide N-acetylgalactosaminyltransferase 6
**31**	88	GSG1L	GSG1 like
**32**	88	HMGA2	high mobility group AT-hook 2
**33**	88	TRABD2B	TraB domain containing 2B
**34**	87	RPL36A- HNRNPH2	RPL36A-HNRNPH2 readthrough
**35**	87	ZNF514	zinc finger protein 514
**36**	87	PHF20L1	PHD finger protein 20 like 1
**37**	87	ADCYAP1R1	ADCYAP receptor type I
**38**	87	RPRML	reprimo like
**39**	86	PRKAR2B	protein kinase cAMP-dependent type II regulatory subunit beta
**40**	86	IBA57	IBA57, iron-sulfur cluster assembly
**41**	86	CSF1	colony stimulating factor 1
**42**	85	SPCS2	signal peptidase complex subunit 2
**43**	85	TENT4B	terminal nucleotidyltransferase 4B
**44**	85	KPNA6	karyopherin subunit alpha 6
**45**	85	GUCY1A1	guanylate cyclase 1 soluble subunit alpha 1
**46**	85	RRM2	ribonucleotide reductase regulatory subunit M2
**47**	84	ZFX	zinc finger protein X-linked
**48**	84	C1orf21	chromosome 1 open reading frame 21
**49**	84	CLMP	CXADR like membrane protein
**50**	84	TMEM100	transmembrane protein 100
**51**	84	MRPL49	mitochondrial ribosomal protein L49
**52**	84	PEA15	proliferation and apoptosis adaptor protein 15
**53**	84	CHKA	choline kinase alpha
**54**	84	WDFY3	WD repeat and FYVE domain containing 3
**55**	84	FAM168A	family with sequence similarity 168 member A
**56**	84	MMP16	matrix metallopeptidase 16
**57**	84	LBH	limb bud and heart development
**58**	83	SUMO1	small ubiquitin-like modifier 1
**59**	83	KMT2A	lysine methyltransferase 2A

**60**	83	LDLRAD4	low density lipoprotein receptor class A domain containing 4
**61**	83	ELOVL3	ELOVL fatty acid elongase 3
**62**	83	EFNA5	ephrin A5
**63**	83	ATP1A2	ATPase Na+/K+ transporting subunit alpha 2
**64**	83	SLITRK2	SLIT and NTRK like family member 2
**65**	83	PSD	pleckstrin and Sec7 domain containing
**66**	82	TSPOAP1	TSPO associated protein 1
**67**	82	ELAVL4	ELAV like RNA binding protein 4
**68**	82	GLP1R	glucagon like peptide 1 receptor
**69**	82	CYB561D1	cytochrome b561 family member D1
**70**	81	NWD1	NACHT and WD repeat domain containing 1
**71**	81	ZGLP1	zinc finger, GATA-like protein 1
**72**	81	PTP4A1	protein tyrosine phosphatase type IVA, member 1
**73**	81	ZBTB10	zinc finger and BTB domain containing 10
**74**	81	TEAD3	TEA domain transcription factor 3
**75**	81	BET1	Bet1 golgi vesicular membrane trafficking protein
**76**	81	CDKL5	cyclin dependent kinase like 5
**77**	81	PDE7A	phosphodiesterase 7A
**78**	81	RBM7	RNA binding motif protein 7
**79**	81	EXOSC3	exosome component 3
**80**	81	CLSPN	Claspin
**81**	80	USP4	ubiquitin specific peptidase 4
**82**	80	PAX5	paired box 5
**83**	80	NDC1	NDC1 transmembrane nucleoporin
**84**	80	MTCH2	mitochondrial carrier 2
**85**	80	GPR182	G protein-coupled receptor 182
**86**	80	TMEM164	transmembrane protein 164
**87**	80	TRPV3	transient receptor potential cation channel subfamily V member 3
**88**	80	IQSEC2	IQ motif and Sec7 domain 2
**89**	80	NEK9	NIMA related kinase 9

**90**	80	TRIM13	tripartite motif containing 13
**91**	80	GOPC	golgi associated PDZ and coiled-coil motif containing
**92**	80	CSF3	colony stimulating factor 3
**93**	80	KDF1	keratinocyte differentiation factor 1
**94**	79	ADIPOR2	adiponectin receptor 2
**95**	79	ST6GALNAC2	ST6N-acetylgalactosaminidealpha-2,6-sialyltransferase 2
**96**	79	PPARGC1A	PPARG coactivator 1 alpha
**97**	79	PIK3CB	phosphatidylinositol-4,5-bisphosphate 3- kinase catalytic subunit beta
**98**	79	BBC3	BCL2 binding component 3
**99**	79	STARD7	StAR related lipid transfer domain containing 7
**100**	79	ZNF680	zinc finger protein 680
**101**	79	IGFL2	IGF like family member 2
**102**	79	PID1	phosphotyrosine interaction domain containing 1
**103**	79	FADS1	fatty acid desaturase 1
**104**	79	ANKRD33B	ankyrin repeat domain 33B
**105**	79	ZNF524	zinc finger protein 524
**106**	79	TARS	threonyl-tRNA synthetase
**107**	79	KIF13A	kinesin family member 13A
**108**	78	SYT2	synaptotagmin 2
**109**	78	TMEM127	transmembrane protein 127
**110**	78	RNF145	ring finger protein 145
**111**	78	B4GALT5	beta-1,4-galactosyltransferase 5
**112**	78	NPTN	Neuroplastin
**113**	78	CNTN5	contactin 5
**114**	78	SMARCD1	SWI/SNF related, matrix associated, actin dependent regulator of chromatin, subfamily d, member 1
**115**	78	ZNF773	zinc finger protein 773
**116**	77	CHD2	chromodomain helicase DNA binding protein 2
**117**	77	UBE2L3	ubiquitin conjugating enzyme E2 L3
**118**	77	MLXIP	MLX interacting protein

**119**	77	PCBP4	poly(rC) binding protein 4
**120**	77	PISD	phosphatidylserine decarboxylase
**121**	77	MCAT	malonyl-CoA-acyl carrier protein transacylase
**122**	77	AMPD2	adenosine monophosphate deaminase 2
**123**	77	ZBTB21	zinc finger and BTB domain containing 21
**124**	77	RAB9A	RAB9A, member RAS oncogene family
**125**	77	ZEB1	zinc finger E-box binding homeobox 1
**126**	77	POLR2H	RNA polymerase II subunit H
**127**	77	MDM4	MDM4, p53 regulator
**128**	77	TRIM11	tripartite motif containing 11
**129**	76	TMEM92	transmembrane protein 92
**130**	76	PERP	PERP, TP53 apoptosis effector
**131**	76	NEDD8	neural precursor cell expressed, developmentally down-regulated 8
**132**	76	SLC30A7	solute carrier family 30 member 7
**133**	76	DAP	death associated protein
**134**	76	LMO7	LIM domain 7
**135**	76	SYT17	synaptotagmin 17
**136**	76	C6orf106	chromosome 6 open reading frame 106
**137**	76	LOC101927844	uncharacterized LOC101927844
**138**	76	STAT1	signal transducer and activator of transcription 1
**139**	76	ZNF718	zinc finger protein 718
**140**	76	OTUD7B	OTU deubiquitinase 7B
**141**	76	RIMKLB	ribosomal modification protein rimK like family member B
**142**	76	ETNK2	ethanolamine kinase 2
**143**	76	AEBP2	AE binding protein 2
**144**	75	TLK2	tousled like kinase 2
**145**	75	CNOT7	CCR4-NOT transcription complex subunit 7
**146**	75	TBC1D16	TBC1 domain family member 16
**147**	75	ZIC4	Zic family member 4
**148**	75	PTK6	protein tyrosine kinase 6

**149**	75	MAPKAPK3	mitogen-activated protein kinase-activated protein kinase 3
**150**	75	ETF1	eukaryotic translation termination factor 1
**151**	75	SMARCD2	SWI/SNF related, matrix associated, actin dependent regulator of chromatin, subfamily d, member 2
**152**	75	NR3C2	nuclear receptor subfamily 3 group C member 2
**153**	75	CBX1	chromobox 1
**154**	75	SNX30	sorting nexin family member 30
**155**	75	ZNF135	zinc finger protein 135
**156**	74	MBD1	methyl-CpG binding domain protein 1
**157**	74	ALPK3	alpha kinase 3
**158**	74	PCNX1	pecanex 1
**159**	74	ZBTB42	zinc finger and BTB domain containing 42
**160**	74	CFLAR	CASP8 and FADD like apoptosis regulator
**161**	74	EIF2B2	eukaryotic translation initiation factor 2B subunit beta
**162**	74	ZBTB7A	zinc finger and BTB domain containing7A
**163**	74	SPOPL	speckle type BTB/POZ protein like
**164**	74	RAPGEF6	Rap guanine nucleotide exchange factor 6
**165**	74	IRGQ	immunity related GTPase Q
**166**	74	SCUBE1	signal peptide, CUB domain and EGF like domain containing 1
**167**	74	GPR26	G protein-coupled receptor 26
**168**	74	CASTOR2	cytosolic arginine sensor for mTORC1 subunit 2
**169**	74	SLC17A4	solute carrier family 17 member 4
**170**	74	SAMD4A	sterile alpha motif domain containing 4A
**171**	73	EFNB1	ephrin B1
**172**	73	RNF141	ring finger protein 141
**173**	73	CALCR	calcitonin receptor
**174**	73	VOPP1	VOPP1, WBP1/VOPP1 family member
**175**	73	CD84	CD84 molecule
**176**	73	ARL6IP1	ADP ribosylation factor like GTPase 6 interacting protein 1

**177**	73	BORA	bora, aurora kinase A activator
**178**	73	PBX1	PBX homeobox 1
**179**	73	PADI1	peptidyl arginine deiminase 1
**180**	73	CNPPD1	cyclin Pas1/PHO80 domain containing 1
**181**	73	CXCL3	C-X-C motif chemokine ligand 3
**182**	73	METTL14	methyltransferase like 14
**183**	72	BAK1	BCL2 antagonist/killer 1
**184**	72	SRGAP3	SLIT-ROBO Rho GTPase activating protein 3
**185**	72	GOLGA8B	golgin A8 family member B
**186**	72	NTRK2	neurotrophic receptor tyrosine kinase 2
**187**	72	RPS6KA2	ribosomal protein S6 kinase A2
**188**	72	RYR3	ryanodine receptor 3
**189**	72	CYP4X1	cytochrome P450 family 4 subfamily X member 1
**190**	72	KIF1A	kinesin family member 1A
**191**	71	ZNF852	zinc finger protein 852
**192**	71	ZNF229	zinc finger protein 229
**193**	71	IPO11	importin 11
**194**	71	NSL1	NSL1, MIS12 kinetochore complex component
**195**	71	CCDC47	coiled-coil domain containing 47
**196**	71	CDK9	cyclin dependent kinase 9
**197**	71	TMEM245	transmembrane protein 245
**198**	71	SLC9B2	solute carrier family 9 member B2
**199**	71	TP53INP1	tumor protein p53 inducible nuclear protein 1
**200**	71	ARRB1	arrestin beta 1
**201**	71	HECTD1	HECT domain E3 ubiquitin protein ligase 1
**202**	71	TRIM47	tripartite motif containing 47
**203**	70	SMAP2	small ArfGAP2
**204**	70	NABP1	nucleic acid binding protein 1
**205**	70	C2orf72	chromosome 2 open reading frame 72
**206**	70	GART	phosphoribosylglycinamide formyltransferase, phosphoribosylglycinamide synthetase, phosphoribosylaminoimidazole synthetase

**207**	70	GOLGA8N	golgin A8 family member N
**208**	70	RPRM	reprimo, TP53 dependent G2 arrest mediator homolog
**209**	70	CELF1	CUGBP Elav-like family member 1
**210**	70	ZKSCAN7	zinc finger with KRAB and SCAN domains 7
**211**	70	ZNF714	zinc finger protein 714
**212**	70	TP53INP2	tumor protein p53 inducible nuclear protein 2
**213**	69	LRFN2	leucine rich repeat and fibronectin type III domain containing 2
**214**	69	RTP1	receptor transporter protein 1
**215**	69	SOD2	superoxide dismutase 2
**216**	69	CCDC68	coiled-coil domain containing 68
**217**	69	ZNF592	zinc finger protein 592
**218**	69	MRPS18A	mitochondrial ribosomal protein S18A
**219**	69	SMC3	structural maintenance of chromosomes 3
**220**	69	CGNL1	cingulin like 1
**221**	69	CHMP2B	charged multivesicular body protein 2B
**222**	69	FAM210B	family with sequence similarity 210 member B
**223**	69	CNIH3	cornichon family AMPA receptor auxiliary protein 3
**224**	68	SYNJ2BP	synaptojanin 2 binding protein
**225**	68	DSG3	desmoglein 3
**226**	68	ZNF440	zinc finger protein 440
**227**	68	ATXN7	ataxin 7
**228**	68	HLA-DOA	major histocompatibility complex, class II, DO alpha
**229**	68	POM121	POM121 transmembrane nucleoporin
**230**	68	TCTEX1D1	Tctex1 domain containing 1
**231**	68	ANKRD63	ankyrin repeat domain 63
**232**	68	NDUFA8	NADH:ubiquinone oxidoreductase subunitA8
**233**	68	IFFO2	intermediate filament family orphan 2
**234**	67	VAV2	vav guanine nucleotide exchange factor 2
**235**	67	FRAT2	FRAT2, WNT signaling pathway regulator
**236**	67	ITGB3	integrin subunit beta 3
**237**	67	ZNF605	zinc finger protein 605

**238**	67	LRRN2	leucine rich repeat neuronal 2
**239**	67	PTBP3	polypyrimidine tract binding protein 3
**240**	67	SPOCK1	SPARC (osteonectin), cwcv and kazal like domains proteoglycan 1
**241**	67	FAM171A1	family with sequence similarity 171 member A1
**242**	67	TRAPPC4	trafficking protein particle complex 4
**243**	67	RSU1	Ras suppressor protein 1
**244**	67	FAM160B1	family with sequence similarity 160 member B1
**245**	67	ORAI2	ORAI calcium release-activated calcium modulator 2
**246**	67	ATP2B1	ATPase plasma membrane Ca2+ transporting 1
**247**	66	SETD5	SET domain containing 5
**248**	66	WNT5B	Wnt family member 5B
**249**	66	MAVS	mitochondrial antiviral signaling protein
**250**	66	KLF15	Kruppel like factor 15
**251**	66	OPA3	OPA3, outer mitochondrial membrane lipid metabolism regulator
**252**	66	ARGFX	arginine-fifty homeobox
**253**	66	UBFD1	ubiquitin family domain containing 1
**254**	66	ATG4B	autophagy related 4B cysteine peptidase
**255**	66	DCUN1D5	defective in cullin neddylation 1 domain containing 5
**256**	65	INPP5A	inositol polyphosphate-5-phosphatase A
**257**	65	METTL7A	methyltransferase like 7A
**258**	65	C2orf68	chromosome 2 open reading frame 68
**259**	65	NIPAL3	NIPA like domain containing 3
**260**	65	DES	Desmin
**261**	65	CALHM5	calcium homeostasis modulator family
**262**	65	ODF2L	outer dense fiber of sperm tails 2 like
**263**	65	NLRP7	NLR family pyrin domain containing 7
**264**	65	CYP26B1	cytochrome P450 family 26 subfamily B member 1
**265**	65	PDIK1L	PDLIM1 interacting kinase 1 like
**266**	65	POU2F1	POU class 2 homeobox 1
**267**	64	LRPAP1	LDL receptor related protein associated protein 1

**268**	64	DNAL4	dynein axonemal light chain 4
**269**	64	TRHDE	thyrotropin releasing hormone degrading enzyme
**270**	64	DTX3L	deltex E3 ubiquitin ligase 3L
**271**	64	FRMD3	FERM domain containing 3
**272**	64	EIF4B	eukaryotic translation initiation factor 4B
**273**	64	TCTN1	tectonic family member 1
**274**	64	CALN1	calneuron 1
**275**	64	HAS3	hyaluronan synthase 3
**276**	64	C9orf152	chromosome 9 open reading frame 152
**277**	64	RFT1	RFT1 homolog
**278**	64	ZHX2	zinc fingers and homeoboxes 2
**279**	64	ASRGL1	asparaginase like 1
**280**	64	ATP8A2	ATPase phospholipid transporting 8A2
**281**	64	ICMT	isoprenylcysteine carboxyl methyltransferase
**282**	64	CEBPA	CCAAT enhancer binding protein alpha
**283**	64	RSRP1	arginine and serine rich protein 1
**284**	64	SEL1L	SEL1L, ERAD E3 ligase adaptor subunit
**285**	64	SMDT1	single-pass membrane protein with aspartate rich tail 1
**286**	64	DUSP13	dual specificity phosphatase 13
**287**	63	ADCY1	adenylate cyclase 1
**288**	63	H6PD	hexose-6-phosphate dehydrogenase/glucose 1-dehydrogenase
**289**	63	ACTRT1	actin related protein T1
**290**	63	TREM1	triggering receptor expressed on myeloid cells 1
**291**	63	FOXO4	forkhead box O4
**292**	63	EIF4G3	eukaryotic translation initiation factor 4 gamma 3
**293**	63	CARM1	coactivator associated arginine methyltransferase 1
**294**	63	TMEM250	transmembrane protein 250
**295**	62	TSEN15	tRNA splicing endonuclease subunit 15
**296**	62	PKP4	plakophilin 4
**297**	62	LEXM	lymphocyte expansion molecule
**298**	62	SNX11	sorting nexin 11

**299**	62	PITPNA	phosphatidylinositol transfer protein alpha
**300**	62	MBTD1	mbt domain containing 1
**301**	62	ATF2	activating transcription factor 2
**302**	62	KCNH8	potassium voltage-gated channel subfamily H member 8
**303**	62	TRAF2	TNF receptor associated factor 2
**304**	62	GNAL	G protein subunit alpha L
**305**	62	RNASEH2C	ribonuclease H2 subunit C
**306**	62	CBL	Cbl proto-oncogene
**307**	62	WSCD2	WSC domain containing 2
**308**	62	LPP	LIM domain containing preferred translocation partner in lipoma
**309**	62	A1CF	APOBEC1 complementation factor
**310**	62	VDR	vitamin D receptor
**311**	62	MYOM3	myomesin 3
**312**	62	UBE2W	ubiquitin conjugating enzyme E2 W
**313**	62	EZH1	enhancer of zeste 1 polycomb repressive complex 2 subunit
**314**	62	BTRC	beta-transducin repeat containing E3 ubiquitin protein ligase
**315**	61	STMN4	stathmin 4
**316**	61	ATP6V1H	ATPase H+ transporting V1 subunit H
**317**	61	MAPT	microtubule associated protein tau
**318**	61	PEG10	paternally expressed 10
**319**	61	CARHSP1	calcium regulated heat stable protein 1
**320**	61	MSTO1	misato mitochondrial distribution and morphology regulator 1
**321**	61	FAM133B	family with sequence similarity 133 member B
**322**	61	HNRNPR	heterogeneous nuclear ribonucleoprotein R
**323**	61	TRIL	TLR4 interactor with leucine rich repeats
**324**	61	CCNJ	cyclin J
**325**	61	CD36	CD36 molecule
**326**	61	SAMD14	sterile alpha motif domain containing 14

**327**	61	PPM1L	protein phosphatase, Mg2+/Mn2+ dependent 1L
**328**	61	USP7	ubiquitin specific peptidase 7
**329**	61	SLCO1A2	solute carrier organic anion transporter family member 1A2
**330**	61	UBE2O	ubiquitin conjugating enzyme E2 O
**331**	61	PIP5K1A	phosphatidylinositol-4-phosphate 5-kinase type 1 alpha
**332**	61	CNTD2	cyclin N-terminal domain containing 2
**333**	61	VHL	von Hippel-Lindau tumor suppressor
**334**	61	TRAFD1	TRAF-type zinc finger domain containing 1
**335**	60	PALD1	phosphatase domain containing paladin 1
**336**	60	UCP3	uncoupling protein 3
**337**	60	CALM3	calmodulin 3
**338**	60	ACOT11	acyl-CoA thioesterase 11
**339**	60	SMIM14	small integral membrane protein 14
**340**	60	CSRNP3	cysteine and serine rich nuclear protein 3
**341**	60	BARX2	BARX homeobox 2
**342**	60	PPP1R3F	protein phosphatase 1 regulatory subunit 3F
**343**	60	AGO1	argonaute RISC catalytic component 1
**344**	60	MAPK4	mitogen-activated protein kinase 4
**345**	60	DIPK2B	divergent protein kinase domain 2B
**346**	60	COL1A1	collagen type I alpha 1 chain
**347**	60	PRTG	Protogenin
**348**	60	SH3TC2	SH3 domain and tetratricopeptide repeats 2
**349**	60	SBNO1	strawberry notch homolog 1
**350**	60	ADPRH	ADP-ribosylarginine hydrolase
**351**	60	ZBTB20	zinc finger and BTB domain containing 20
**352**	60	GRAP2	GRB2 related adaptor protein 2
**353**	60	USH1G	USH1 protein network component sans
**354**	59	IST1	IST1, ESCRT-III associated factor
**355**	59	GIMAP6	GTPase, IMAP family member 6
**356**	59	SOCS7	suppressor of cytokine signaling 7
**357**	59	IGF2BP1	insulin like growth factor 2 mRNA binding protein 1

**358**	59	RNF135	ring finger protein 135
**359**	59	CMTM6	CKLF like MARVEL transmembrane domain containing 6
**360**	59	RAD54L	RAD54 like
**361**	59	MLPH	Melanophilin
**362**	59	AADACL3	arylacetamide deacetylase like 3
**363**	59	ADAM8	ADAM metallopeptidase domain 8
**364**	59	MOB1B	MOB kinase activator 1B
**365**	59	AXDND1	axonemal dynein light chain domain containing 1
**366**	59	CD99	CD99 molecule (Xg blood group)
**367**	59	PIK3R1	phosphoinositide-3-kinase regulatory subunit 1
**368**	59	NRXN3	neurexin 3
**369**	59	CIAO3	cytosolic iron-sulfur assembly component 3
**370**	59	HCN3	hyperpolarization activated cyclic nucleotide gated potassium channel 3
**371**	59	PERM1	PPARGC1 and ESRR induced regulator, muscle 1
**372**	59	DKC1	dyskerin pseudouridine synthase 1
**373**	59	ANKRD34A	ankyrin repeat domain 34A
**374**	59	ST6GAL1	ST6 beta-galactoside alpha-2,6- sialyltransferase 1
**375**	58	SLC35G3	solute carrier family 35 member G3
**376**	58	P2RX6	purinergic receptor P2X 6
**377**	58	ST7L	suppression of tumorigenicity 7 like
**378**	58	MYPOP	Myb related transcription factor, partner of profilin
**379**	58	ATPSCKMT	ATP synthase c subunit lysine N- methyltransferase
**380**	58	INO80D	INO80 complex subunit D
**381**	58	PPM1D	protein phosphatase, Mg2+/Mn2+ dependent 1D
**382**	58	JPH3	junctophilin 3
**383**	58	FOXR2	forkhead box R2
**384**	58	CTNNA3	catenin alpha 3
**385**	58	TNFRSF14	TNF receptor superfamily member 14
**386**	58	CYP3A4	cytochrome P450 family 3 subfamily A member 4
**387**	58	SLC25A42	solute carrier family 25 member 42

**388**	58	PIP4K2B	phosphatidylinositol-5-phosphate 4-kinase type 2 beta
**389**	58	GPATCH2L	G-patch domain containing 2 like
**390**	58	ASPN	Asporin
**391**	58	MGAT5	alpha-1,6-mannosylglycoprotein 6-beta-N-acetylglucosaminyltransferase
**392**	58	RAD51	RAD51 recombinase
**393**	58	R3HCC1L	R3H domain and coiled-coil containing 1 like
**394**	58	GALNT1	polypeptide N-acetylgalactosaminyltransferase 1
**395**	58	PPP1R1A	protein phosphatase 1 regulatory inhibitor subunit 1A
**396**	58	ADORA1	adenosine A1 receptor
**397**	57	DDTL	D-dopachrome tautomerase like
**398**	57	TNS1	tensin 1
**399**	57	CD38	CD38 molecule
**400**	57	C6orf132	chromosome 6 open reading frame 132
**401**	57	SOGA3	SOGA family member 3
**402**	57	MYSM1	Myb like, SWIRM and MPN domains 1
**403**	57	HHIPL2	HHIP like 2
**404**	57	SLC1A3	solute carrier family 1 member 3
**405**	57	AMOTL2	angiomotin like 2
**406**	57	CPD	carboxypeptidase D
**407**	57	UNKL	unk like zinc finger
**408**	57	MAFK	MAF bZIP transcription factor K
**409**	57	PSAPL1	prosaposin like 1 (gene/pseudogene)
**410**	57	TMEM252	transmembrane protein 252
**411**	57	XRN1	5'-3' exoribonuclease 1
**412**	57	PTGER3	prostaglandin E receptor 3
**413**	57	TNC	tenascin C
**414**	57	HIPK2	homeodomain interacting protein kinase 2
**415**	57	SUPT3H	SPT3 homolog, SAGA and STAGA complex component
**416**	56	NR1D2	nuclear receptor subfamily 1 group D member 2
**417**	56	IPO9	importin 9

**418**	56	ZNF500	zinc finger protein 500
**419**	56	PLXNA4	plexin A4
**420**	56	PSD3	pleckstrin and Sec7 domain containing 3
**421**	56	TMEM229B	transmembrane protein 229B
**422**	56	SPTLC2	serine palmitoyltransferase long chain base subunit 2
**423**	56	RND2	Rho family GTPase 2
**424**	56	AMOTL1	angiomotin like 1
**425**	56	FOSL2	FOS like 2, AP-1 transcription factor subunit
**426**	56	TLE3	TLE family member 3, transcriptional corepressor
**427**	56	KCNIP1	potassium voltage-gated channel interacting protein 1
**428**	56	MEDAG	mesenteric estrogen dependent adipogenesis
**429**	56	TADA1	transcriptional adaptor 1
**430**	56	SKAP2	src kinase associated phosphoprotein 2
**431**	56	CACNG8	calcium voltage-gated channel auxiliary subunit gamma 8
**432**	56	THSD7A	thrombospondin type 1 domain containing 7A
**433**	56	NRBF2	nuclear receptor binding factor 2
**434**	56	SLC49A4	solute carrier family 49 member 4
**435**	56	GOLGA8A	golgin A8 family member A
**436**	56	MRPS25	mitochondrial ribosomal protein S25
**437**	56	SAP30BP	SAP30 binding protein
**438**	55	HSPB7	heat shock protein family B (small) member 7
**439**	55	NGB	Neuroglobin
**440**	55	GOLGA8J	golgin A8 family member J
**441**	55	ING4	inhibitor of growth family member 4
**442**	55	TMEM181	transmembrane protein 181
**443**	55	GOLGA8H	golgin A8 family member H
**444**	55	SREBF2	sterol regulatory element binding transcription factor 2
**445**	55	ZNF829	zinc finger protein 829
**446**	55	CNBP	CCHC-type zinc finger nucleic acid binding protein
**447**	55	GDAP2	ganglioside induced differentiation associated protein 2
**448**	55	ARHGEF10L	Rho guanine nucleotide exchange factor 10 like

**449**	55	ZNF74	zinc finger protein 74
**450**	55	PLXNA2	plexin A2
**451**	55	CELSR3	cadherin EGF LAG seven-pass G-type receptor 3
**452**	55	SP1	Sp1 transcription factor
**453**	55	GNL1	G protein nucleolar 1 (putative)
**454**	55	KSR2	kinase suppressor of ras 2
**455**	55	GOLGA8O	golgin A8 family member O
**456**	55	GOLGA8R	golgin A8 family member R
**457**	55	TBC1D2B	TBC1 domain family member 2B
**458**	55	NLK	nemo like kinase
**459**	55	ARFIP2	ADP ribosylation factor interacting protein 2
**460**	55	HCN4	hyperpolarization activated cyclic nucleotide gated potassium channel 4
**461**	55	MTMR12	myotubularin related protein 12
**462**	55	PPP1R13B	protein phosphatase 1 regulatory subunit 13B
**463**	55	NCR3	natural cytotoxicity triggering receptor 3
**464**	55	UBE2D3	ubiquitin conjugating enzyme E2 D3
**465**	55	GK	glycerol kinase
**466**	55	GOLGA8K	golgin A8 family member K
**467**	55	ZNF554	zinc finger protein 554
**468**	55	GRK6	G protein-coupled receptor kinase 6
**469**	55	RLF	rearranged L-myc fusion
**470**	54	PDE7B	phosphodiesterase 7B
**471**	54	WFDC1	WAP four-disulfide core domain 1
**472**	54	SHPK	sedoheptulokinase
**473**	54	HNF4A	hepatocyte nuclear factor 4 alpha
**474**	54	STARD9	StAR related lipid transfer domain containing 9
**475**	54	RASD2	RASD family member 2
**476**	54	THAP3	THAP domain containing 3
**477**	54	UBE2E1	ubiquitin conjugating enzyme E2 E1
**478**	54	KIAA1147	KIAA1147
**479**	54	CA8	carbonic anhydrase 8

**480**	54	RAB3B	RAB3B, member RAS oncogene family
**481**	54	KCNAB1	potassium voltage-gated channel subfamily A member regulatory beta subunit 1
**482**	54	DIRAS2	DIRAS family GTPase 2
**483**	54	TNFSF11	TNF superfamily member 11
**484**	54	MYORG	myogenesis regulating glycosidase (putative)
**485**	54	PSMB6	proteasome subunit beta 6
**486**	54	MGST2	microsomal glutathione S-transferase 2
**487**	54	NEURL1B	neuralized E3 ubiquitin protein ligase 1B
**488**	54	ZNF776	zinc finger protein 776
**489**	53	DNAJB5	DnaJ heat shock protein family (Hsp40) member B5
**490**	53	GOLGA6L9	golgin A6 family-like 9
**491**	53	DLG3	discs large MAGUK scaffold protein 3
**492**	53	GOLGA6L4	golgin A6 family-like 4
**493**	53	MGAT3	mannosyl (beta-1,4-)-glycoprotein beta-1,4-N-acetylglucosaminyltransferase
**494**	53	LTN1	listerin E3 ubiquitin protein ligase 1
**495**	53	UBE2H	ubiquitin conjugating enzyme E2 H
**496**	53	ZNF736	zinc finger protein 736
**497**	53	CTIF	cap binding complex dependent translation initiation factor
**498**	53	PRR3	proline rich 3
**499**	53	ZNF775	zinc finger protein 775
**500**	53	GPRC5A	G protein-coupled receptor class C group 5 member A
**501**	53	TMEM101	transmembrane protein 101
**502**	53	APC2	APC2, WNT signaling pathway regulator
**503**	53	AMZ1	archaelysin family metallopeptidase 1
**504**	53	RRAD	RRAD, Ras related glycolysis inhibitor and calcium channel regulator
**505**	53	RAB12	RAB12, member RAS oncogene family
**506**	53	RAB11FIP4	RAB11 family interacting protein 4
**507**	52	TEAD1	TEA domain transcription factor 1

**508**	52	OSCAR	osteoclast associated, immunoglobulin-like receptor
**509**	52	TRIM71	tripartite motif containing 71
**510**	52	SAMD12	sterile alpha motif domain containing 12
**511**	52	UBE2E3	ubiquitin conjugating enzyme E2 E3
**512**	52	NLN	Neurolysin
**513**	52	MEPCE	methylphosphate capping enzyme
**514**	52	UMPS	uridine monophosphate synthetase
**515**	52	BRF2	BRF2, RNA polymerase III transcription initiation factor subunit
**516**	52	BRF1	BRF1, RNA polymerase III transcription initiation factor subunit
**517**	52	SLC15A2	solute carrier family 15 member 2
**518**	52	GGNBP2	gametogenetin binding protein 2
**519**	52	NUP93	nucleoporin 93
**520**	52	PHKG1	phosphorylase kinase catalytic subunit gamma 1
**521**	52	LIMK2	LIM domain kinase 2
**522**	52	MEFV	MEFV, pyrin innate immunity regulator
**523**	52	RASSF2	Ras association domain family member 2
**524**	52	JCAD	junctional cadherin 5 associated
**525**	52	ZDHHC8	zinc finger DHHC-type containing 8
**526**	52	SLC38A3	solute carrier family 38 member 3
**527**	52	HOXA11	homeobox A11
**528**	52	PPP1R2	protein phosphatase 1 regulatory inhibitor subunit 2
**529**	52	NSUN4	NOP2/Sun RNA methyltransferase family member 4
**530**	51	NFAM1	NFAT activating protein with ITAM motif1
**531**	51	ACVRL1	activin A receptor like type 1
**532**	51	GRHL2	grainyhead like transcription factor 2
**533**	51	SHISAL1	shisa like 1
**534**	51	MCFD2	multiple coagulation factor deficiency 2
**535**	51	ADAM22	ADAM metallopeptidase domain 22
**536**	51	GATAD2B	GATA zinc finger domain containing 2B
**537**	51	TMEM199	transmembrane protein 199

**538**	51	PIRT	phosphoinositide interacting regulator of transient receptor potential channels
**539**	51	SLC25A30	solute carrier family 25 member 30
**540**	51	PRRT2	proline rich transmembrane protein 2
**541**	51	FAM76A	family with sequence similarity 76 member A
**542**	51	RAPGEF1	Rap guanine nucleotide exchange factor 1
**543**	51	CLDN2	claudin 2
**544**	51	TBC1D22B	TBC1 domain family member 22B
**545**	51	AZGP1	alpha-2-glycoprotein 1, zinc-binding
**546**	51	FOXK1	forkhead box K1
**547**	51	AGO4	argonaute RISC catalytic component 4
**548**	51	SH3PXD2A	SH3 and PX domains 2A
**549**	50	STYX	serine/threonine/tyrosine interacting protein
**550**	50	CDKN1B	cyclin dependent kinase inhibitor 1B
**551**	50	LMAN2L	lectin, mannose binding 2 like
**552**	50	CACNA2D4	calcium voltage-gated channel auxiliary subunit alpha2delta 4
**553**	50	ERMN	Ermin
**554**	50	FAM124A	family with sequence similarity 124 member A
**555**	50	THBS1	thrombospondin 1
**556**	50	CCSAP	centriole, cilia and spindle associated protein
**557**	50	C11orf71	chromosome 11 open reading frame 71
**558**	50	MTA3	metastasis associated 1 family member 3
**559**	50	OASL	2'-5'-oligoadenylate synthetase like
**560**	50	UNC119	unc-119 lipid binding chaperone
**561**	50	RAPH1	Ras association (RalGDS/AF-6) and pleckstrin homology domains 1
**562**	50	FAM168B	family with sequence similarity 168 member B
**563**	50	SLC33A1	solute carrier family 33 member 1
**564**	50	UCP2	uncoupling protein 2
**565**	50	SYNCRIP	synaptotagmin binding cytoplasmic RNA interacting protein

**566**	50	NYAP1	neuronal tyrosine phosphorylated phosphoinositide-3-kinase adaptor 1
**567**	50	TBC1D13	TBC1 domain family member 13
**568**	50	S100PBP	S100P binding protein

Supplementary Table-4. Differentially expressed genes in sarcopenia samples

Gene	Gene Description	Change fold	P Value	Expression
IL1B	Interleukin-1 beta	14.5	0.00032	Upregulation
S100A8	S100 calcium binding protein A8	13.4	0.00054	Upregulation
NFKB1	Nuclear factor Kappa B subunit 1	12.9	0.00067	Upregulation
IL6	Interleukin-6	12.2	0.00078	Upregulation
TNFA	Tumor necrosis factor alpha	11.9	0.00031	Upregulation
SOD1	Superoxide dismutase 1	11.5	0.00028	Upregulation
PTGS1	Prostaglandin-endoperoxide synthase 1	11.3	0.00065	Upregulation
BAK1	BCL2 antagonist/killer 1	10.9	0.00068	Upregulation
CD40	CD40 molecyle	10.8	0.00077	Upregulation
CXCL10	C-X-C motif chemokine ligand 10	10.7	0.00098	Upregulation
TGFB1	Transforming growth factor beta 1	10.5	0.00032	Upregulation
ICAM1	Intercellular adhesion molecule 1	10.3	0.00015	Upregulation
S100A9	S100 calcium binding protein A9	10.2	0.00035	Upregulation
VCAM1	Vascular cell adhesion molecule 1	9.9	0.00046	Upregulation
NLRP1	NLR family pyrin domain containing 1	9.5	0.00041	Upregulation
NLRP3	NLR family pyrin domain containing 3	9.2	0.00053	Upregulation
CASP1	Caspase 1	8.9	0.00064	Upregulation
CASP3	Caspase 3	8.5	0.00072	Upregulation
MDM2	MDM2 Proto-oncogene	8.3	0.00093	Upregulation
DUSP2	Dual specificity phosphatase 2	7.8	0.00065	Upregulation
CXCL2	C-X-C motif chemokine ligand 2	7.4	0.00035	Upregulation
SOD2	Superoxide dismutase 2	7.2	0.00064	Upregulation
PTGS2	Prostaglandin-endoperoxide synthase 2	7.2	0.00067	Upregulation
MMP13	Matrix metallopeptidase 13	7.1	0.00034	Upregulation
INFG	Interferon gamma	7.0	0.00065	Upregulation
TRAF6	TNF receptor associated factor 6	7.0	0.00011	Upregulation

BBC3	BCL2 binding component 3 MYC Proto-oncogene, BHLH transcription factor	6.9	0.00037	Upregulation
ZBTB9	Zinc finger and BTB Domain containing 9	6.7	0.00056	Upregulation
VPS52	Vacuolar protein sorting 52 homolog	6.5	0.00044	Upregulation
ZNF514	Zinc finger Protein 514	6.2	0.00036	Upregulation
SGK1	Serum/Glucocorticoid regulated kinase 1	5.9	0.00064	Upregulation
CAPN2	Calpain 2	5.7	0.00016	Upregulation
LTA	Lymphotoxin alpha	5.4	0.00053	Upregulation
GZMB	Granzyme B	5.3	0.00025	Upregulation
TRAF3	TNF receptor associated factor	5.1	0.00065	Upregulation
ITPR1	Inositol 1,4,5-Trisphosphate receptor type 1	5.1	0.00011	Upregulation
GRM4	Glutamate metabotropic receptor 4	4.9	0.00026	Upregulation
NUDT3	Nudix hydrolase 3	4.6	0.00092	Upregulation
CDC42	Cell division cycle 42	4.5	0.00079	Upregulation
PEA15‘	Proliferation and apoptosis adaptor protein 15	3.9	0.00084	Upregulation
NOL3	Nucleolar protein 3	3.7	0.00064	Upregulation
DDB2	Damage specific DNA binding protein 2	3.6	0.00028	Upregulation
CCND3	Cyclin D3	3.4	0.00017	Upregulation
MFN2	Mitofusin 2	3.3	0.00036	Upregulation
AXIN2	Axin 2	3.1	0.00034	Upregulation
DDB1	Damage specific DNA binding protein 2	2.9	0.00022	Upregulation
LEF1	Lymphoid enhancer binding factor 1	2.7	0.00012	Upregulation
PSEN2	Presenilin 2	2.5	0.00055	Upregulation
ANK1	Ankyrin 1	2.4	0.00017	Upregulation
CDH1	Cadherin 1	-15.1	0.0013	Downregulation

BDH1	3-Hydroxybutyrate dehydrogenase 1	-14.2	0.00018	Downregulation
PON1	Paraoxonase 1	-13.9	0.0091	Downregulation
BDH2	3-Hydroxybutyrate dehydrogenase 2	-13.4	0.00054	Downregulation
CD117	CD117 molecule	-12.7	0.0032	Downregulation
BMP4	Bone morphogenetic protein 4	-12.3	0.0065	Downregulation
GNG11	G Protein subunit gamma 11	-11.6	0.00043	Downregulation
NPTX1	Neuronal pentraxin 1	-11.2	0.00074	Downregulation
PTX3	Pentraxin 3	-10.3	0.00061	Downregulation
ZAP70	Zeta chain of T cell receptor associated protein kinase 70	-9.8	0.00093	Downregulation
SLA2	Src like adaptor 2	-8.2	0.0024	Downregulation
NR3C1	Nuclear receptor subfamily 3 group C member 1	-7.8	0.0018	Downregulation
CST3	Cystatin C	-7.4	0.00057	Downregulation
FGF9	Fibroblast growth factor 9	-7.1	0.00015	Downregulation
MFSD7	Major facilitator superfamily domain-containing protein 7	-6.7	0.00046	Downregulation
GLTPD	Glycolipid transfer protein domain containing 1	-6.4	0.0063	Downregulation
LRG1	Leucine-rich alpha-2-glycoprotein 1	-5.3	0.00074	Downregulation
ZFPM1	Zinc finger protein, FOG family member 1	-4.5	0.0084	Downregulation
KIF2A	Kinesin family member 2A	-4.2	0.00065	Downregulation
SORT1	Sortilin 1	-3.4	0.00043	Downregulation
INTS12	Integrator complex subunit 12	-2.5	0.00024	Downregulation
